# Reactivation of encoding ensembles in the prelimbic cortex supports temporal associations

**DOI:** 10.1038/s41386-024-01825-2

**Published:** 2024-03-07

**Authors:** Thays Brenner Santos, Cesar Augusto de Oliveira Coelho, Juliana Carlota Kramer-Soares, Paul W. Frankland, Maria Gabriela Menezes Oliveira

**Affiliations:** 1 Departamento de Psicobiologia, Universidade Federal de São Paulo - UNIFESP, São Paulo, 04023-062 Brazil; 2 Neuroscience and Mental Health, Hospital for Sick Children, Toronto, ON M5G 0A4 Canada; 3 Universidade Cruzeiro do Sul - UNICSUL, São Paulo, 08060-070 Brazil; 4 Department of Physiology, University of Toronto, Toronto, ON M5G 1X8 Canada; 5 Department of Psychology, University of Toronto, Toronto, ON M5G 1X8 Canada; 6 Institute of Medical Sciences, University of Toronto, Toronto, ON M5G 1X8 Canada; 7 Child & Brain Development Program, Canadian Institute for Advanced Research, Toronto, ON M5G 1M1 Canada

**Keywords:** Fear conditioning, Prefrontal cortex

## Abstract

Fear conditioning is encoded by strengthening synaptic connections between the neurons activated by a conditioned stimulus (CS) and those activated by an unconditioned stimulus (US), forming a memory engram, which is reactivated during memory retrieval. In temporal associations, activity within the prelimbic cortex (PL) plays a role in sustaining a short-term, transient memory of the CS, which is associated with the US after a temporal gap. However, it is unknown whether the PL has only a temporary role, transiently representing the CS, or is part of the neuronal ensembles that support the retrieval, i.e., whether PL neurons support both transient, short-term memories and stable, long-term memories. We investigated neuronal ensembles underlying temporal associations using fear conditioning with a 5-s interval between the CS and US (CFC-5s). Controls were trained in contextual fear conditioning (CFC), in which the CS-US overlaps. We used Robust Activity Marking (RAM) to selectively manipulate PL neurons activated by CFC-5s learning and Targeted Recombination in Active Populations (TRAP2) mice to label neurons activated by CFC-5s learning and reactivated by memory retrieval in the amygdala, medial prefrontal cortex, hippocampus, perirhinal cortices (PER) and subiculum. We also computed their co-reactivation to generate correlation-based networks. The optogenetic reactivation or silencing of PL encoding ensembles either promoted or impaired the retrieval of CFC-5s but not CFC. CFC-5s retrieval reactivated encoding ensembles in the PL, PER, and basolateral amygdala. The engram network of CFC-5s had higher amygdala and PER centralities and interconnectivity. The same PL neurons support learning and stable associative memories.

## Introduction

Linking information across time is essential for survival. We rely on past information about space and objects to navigate dynamic environments, find resources, and avoid danger. Transient memories of past stimuli associate events that occur separately but close together in time, known as temporal associations [1, 2]. Importantly, dysfunction in retaining or linking transient memories can lead to memory deficits and maladaptive behaviors inflexible to changes. In models of Alzheimer’s disease, schizophrenia, or aging, rodents exhibit impairment in temporal associations [3–5].

Conceptualizations have proposed that memories are encoded by strengthening synaptic connections between the neurons activated during the experience, forming a memory engram in a distributed brain network [6–8]. Restoring activity in this same neuronal ensemble is sufficient to induce memory retrieval. During trace fear conditioning (tFC), a temporal association in which the conditioned stimulus (CS) is separated in time from the unconditioned stimulus (US), prelimbic cortex (PL) neurons exhibit sustained firing during the CS-US interval [9–11]. Using optogenetics to inactivate PL neurons precisely during this interval impairs memory formation, suggesting that PL supports CS representations during the interval [12].

However, it is unknown whether the same population of PL neurons that support learning also support memory retrieval, i.e., whether their inhibition can block retrieval and their reactivation induces retrieval. It is also unknown whether other regions are part of the encoding and retrieval ensembles of temporal associations and how they are organized at the network level. Prior studies have mainly investigated the participation of single regions in one memory phase (learning or retrieval) [1].

Only studies examining associations of stimuli that overlapped in time, such as tone or contextual fear conditioning (CFC), have investigated PL encoding ensembles in retrieval. They showed that PL neurons are activated by both learning and retrieval [7, 13]. These PL encoding ensembles reorganize over time to support remote memories, being distinct from those activated by learning [14, 15]. So far, only studies targeting the mPFC have investigated the necessity of PL encoding ensembles in recent memories [16, 17]. The PL can be dispensable to encode tone or CFC [18–21] but see [22–24], although it shows learning-related activation and plasticity following fear conditioning [25–28]. In turn, the PL is essential for memory retrieval of tone and CFC, showing increased responses to the CS in the test sessions [10, 18, 29–33]. When associations need PL-dependent functions, such as transient memories, PL encoding ensembles may become necessary for retrieval from recent post-learning times.

Besides transient memories and PL activity, temporal associations may involve additional processes and their neurobiological correlates, such as attention, tracking timing, interference reduction, US expectancy, and changes in associative strength [1, 2, 34]. Different theoretical mechanisms may change the neurobiology of learning, reorganizing regions globally [34]. Investigating how multiple regions co-activate may reveal connectivity differences reflecting this reorganization.

We investigated encoding and retrieval ensembles of temporal associations in individual regions and correlation-based networks in observational, gain-of-function, and loss-of-function experiments. Using CFC-5s, in which a contextual CS is separated by a 5-s interval from the US [20, 35–37], we evaluated opto-reactivating or silencing PL encoding ensembles on CFC-5s retrieval. We also investigated the reactivation of encoding ensembles by retrieval in the medial prefrontal cortex (mPFC), amygdala, hippocampus, and parahippocampal area (PH), as activation in these areas accompanied CFC-5s learning [37]. We used their co-reactivation to generate putative networks and graph theory methods to analyze them.

## Materials and methods

### Subjects

We used 166 C57BL/6NTac x 29S6/SvEvTac wild-type mice and 19 c-fos-CRE-ERT2-v2-TdTomato (TRAP2) mice of 8–12 weeks old from the Hospital for Sick Children (SickKids), with a similar number of male and female in each group. The mice were housed in groups of 4 at a controlled temperature (22 °C ± 1 °C), on a 12-h light-dark cycle, with food and water ad libitum, and were acclimatized for one week. In optogenetic experiments, C57BL/6 N WT mice were maintained on doxycycline (DOX) chow (40 mg/kg). All procedures followed the policies of the Canadian Council on Animal Care and were approved by the SickKids Committee. Sample sizes were estimated on G*Power [38].

### Apparatus

The conditioning chamber comprised a 31 × 24 × 21 cm aluminum and acrylic box with a shock grid (Med Associates). Context 1 was a 30 × 35 cm white cylinder, and Context 2 was a triangular white box inside the conditioning chamber. The opto-stimulation context was a 22 × 35 × 20 cm cage. The transition cage was the opto-stimulation context with corncob bedding on the floor and accommodated the mice during the 15 s to 60 min intervals. Video cameras recorded all sessions.

### Characterization of CFC-5s

**Fig. 1:**
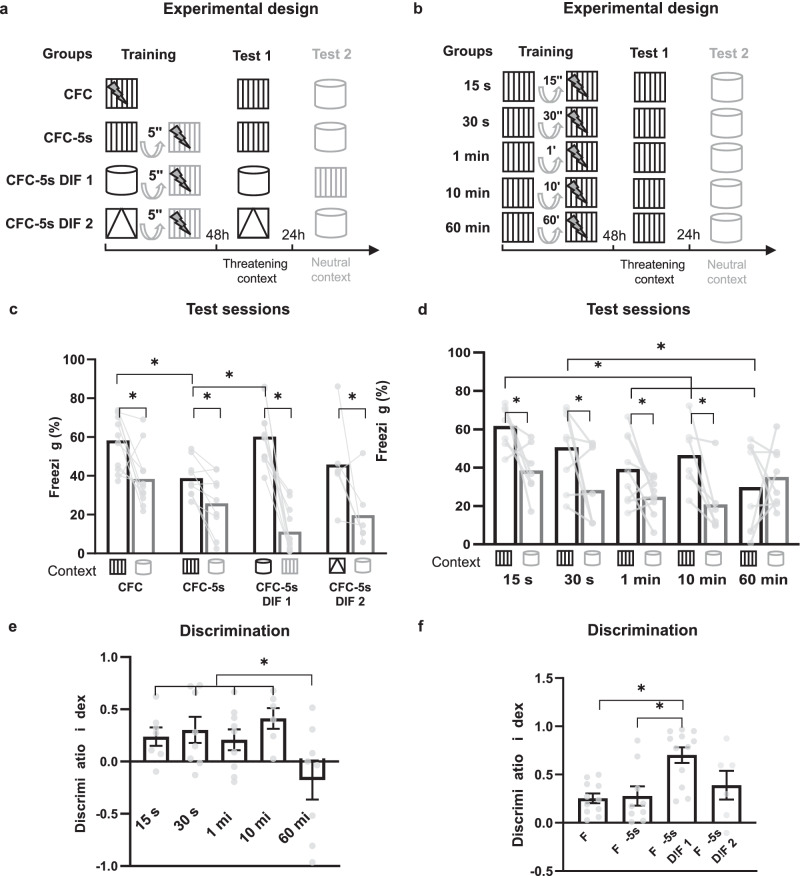
The context is associated separated in time from the US up to 10-minute intervals. **a**, **b** Experimental designs. **c**, **d** Mean (±standard error) of freezing time in the threatening and neutral contexts in the test sessions. **e**, **f** Mean (±standard error) of the discrimination index. * Indicates *p* < 0.050. Generalized Estimating Equation or Generalized Linear Models followed by LSD test. Dots show sample distribution. CFC contextual fear conditioning, CFC-5s CFC with a 5-s interval, CFC-5s DIF CFC-5s using different contexts as the CS and US’s background.

We trained three groups of mice in CFC-5s, which had a 5-s interval between the contextual CS and US, and one group in traditional CFC without intervals. We performed two experimental designs for the CFC-5s task to investigate the effect of different contexts used as the CS and the background during the immediate US. For the CFC-5s group, the threatening context (the contextual CS before the interval) and the context of the US background were the same. For the CFC-5s, different (DIF) groups contexts were different. We trained two CFC-5s DIF groups (CFC-5s DIF 1 and CFC-5s DIF 2), using different threatening contexts to verify if the results were context-independent. C57BL/6 N WT mice were handled for 5 min for three consecutive days to habituate. In the training, we exposed them for 5 min to the conditioning chamber (CFC-5s *n* = 9), Context 1 (CFC-5s DIF 1), or Context 2 (CFC-5s DIF, 2 *n* = 6). These were considered threatening contexts. We held them for a 5-s interval and placed them in the conditioning chamber, delivering one immediate footshock (1 mA, 2 s). The control group for associations overlapped in time (CFC group, *n* = 11) was exposed to the conditioning chamber for 5 min, receiving one footshock at the end. We tested all mice 48 and 72 h later in the threatening context or a neutral context for 5 min, counterbalancing the context order (Fig. 1a).

We also investigated the effect of the interval length on memory specificity. Using matching contexts, we varied the CS-US interval from 15 s to 60 min. We trained C57BL/6 N WT mice in CFC with time intervals between the threatening context (conditioning chamber) and the US of 15 s (*n* = 7), 30 s (*n* = 8), 1 min (*n* = 9), 10 min (*n* = 6) or 60 min (*n* = 8). We tested them 48 and 72 h later in the threatening context or neutral context, counterbalanced (Fig. 1b). Freezing was measured by FreezeFrame (Actimetrics, V3.32). Freezing to the threatening context was considered context-specific and to a neutral context unspecific.

### Activity-dependent virus expressing opsins

We used AVV(DJ)-RAM-ChR2, AVV(DJ)-RAM-NpACY, or AVV(DJ)-RAM-GFP viruses. RAM combines activity-dependent promoters in a modified Tetracycline-Off system. Without the DOX, activated neurons express the effector genes (ChR2, NpACY, or GFP) [39]. The AVV(DJ)-RAM-ChR2 expresses enhanced Channelrhodopsin 2 (ChR2/H134R), which allows cation influx resulting in depolarization. The AVV(DJ)-RAM-NpACY expresses both ChR2 and halorhodopsin 3.0 (NpHR3.0), which pumps chloride ions resulting in hyperpolarization. The NpACY construct enables bidirectional control of neuronal activity by non-overlapping light wavelengths, although we only used it for hyperpolarization in silencing experiments. The AAV-RAM-d2TTA::TRE-EGFP-WPREpA was a gift from Yingxi Lin (Addgene #84469). The AAV-(DJ) was generated using the AAV-DJ Helper Free Packaging System (VPK-400-DJ, Cell Biolabs) in HEK293T cells. The final viral titers were approximately 10^11^/ml. All viruses were stored at −80 °C until use.

### Viral vector injection and optrode implant

We anesthetized mice with intraperitoneal (IP) injections of Chloral Hydrate (400 mg/kg) and Atropine Sulfate (0.1 mg/kg) and fixed them in the stereotaxic frame. We inject 0.3 μl per side of one of the viruses into the PL (anteroposterior +1.9 mm, mediolateral ±0.3 mm, and dorsoventral −2.4 mm from bregma) [40] at 0.1 μl/min using a glass pipette attached to a micro-syringe. The pipettes stayed on for 10 min. We implanted one optical fiber (200 µm, 0.22 NA, 10.0 mm) assembled to a ceramic ferrule (Ø1.25 mm, 6.4 mm) above the PL (mediolateral 0 mm, dorsoventral −2.1 mm). Mice received post-surgery subcutaneous injections of Meloxicam (2 mg/kg) and recovered for 21 days.

### Effects of reactivating or silencing PL encoding ensembles on memory retrieval

**Fig. 2:**
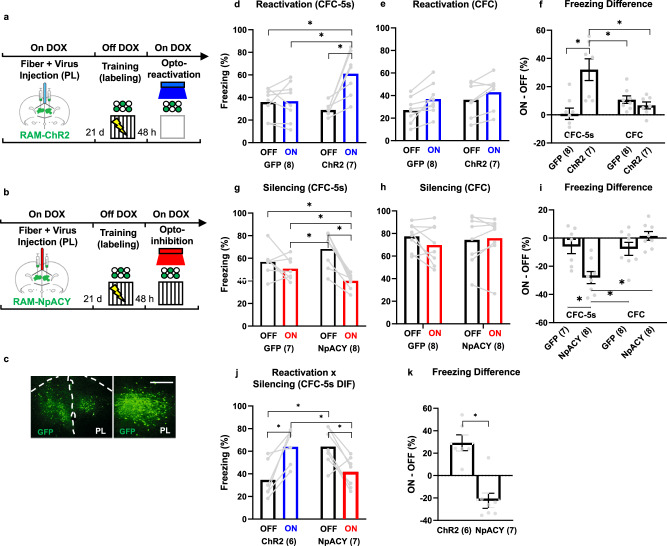
Encoding ensembles in the PL induced and were necessary for memory retrieval of associations separated, but not overlapped, in time. **a**, **b** Experimental designs. **c** Representative image of viral expression (GFP-positive cells) in the PL. **d**–**f** Reactivation of PL ensembles in mice trained in CFC-5s and CFC. **g**–**i** Silencing of PL ensembles in mice trained in CFC-5s and CFC. **j**–**k** Reactivation or silencing of PL ensembles in mice trained in CFC-5s DIF. Mean ± standard error of the freezing time during the lights OFF and ON periods or the mean difference of freezing time during the light ON and OFF periods. * Indicates *p* < 0.050. Generalized Estimating Equations or Generalized Linear Models followed by LSD test. Dots show sample distribution. See training and habituation sessions in Supplementary Fig. 1 and the percentage of infected cells in Supplementary Fig. 2. ChR2 Channelrhodopsin 2, DOX doxycycline, GFP green fluorescent protein, NpACY construct with ChR2 + NpHR3.0 (halorhodopsin 3.0), PL prelimbic cortex, RAM Robust Activity Marking. See group names in Fig. 1.

C57BL/6 N WT mice injected with AVV(DJ)-RAM-ChR2, AVV(DJ)-RAM-NpACY, or AVV(DJ)-RAM-GFP were habituated for three days. On the third day, they were also habituated to the optogenetic procedures, receiving blue or red light in the opto-stimulation context for 4 min, and put off the DOX diet. They were trained 48 h later in the CFC-5s, CFC, or CFC-5s DIF, returning to the DOX diet immediately after training. Without DOX, PL neurons activated by training were labeled with ChR2 or GFP via the RAM system. Forty-eight hours later, PL encoding ensembles were either reactivated in a neutral context or silenced in the threatening context. For this, mice were put in the opto-stimulation context for 8 min. After a 4-min baseline (lights OFF), blue light stimulation (lights ON, 473 nm; 4-Hz; 15-ms pulses; 1–2 mW) was delivered to promote the reactivation of PL encoding ensembles. In silencing experiments, mice were tested in their threatening context (conditioning chamber for CFC-5s and CFC groups and Context 1 for CFC-5s DIF group) for 6 min. After a 3-min baseline (lights OFF), red light stimulation (lights ON, 595 nm; 10-Hz; constant; 10 mW) was delivered for 3 min to promote inhibition of PL encoding ensembles. Freezing was measured continuously with a stopwatch (Fig. 2a, b).

### Tamoxifen

We dissolved 30 mg of tamoxifen (T5648, Sigma-Aldrich) in 100 μl of 100% ethanol and 900 μl of sunflower oil (30 mg/ml) and injected 180 mg/kg IP 24 h before training [14, 41, 42]. TRAP2 mice express the transgene iCreERT2 from a c-Fos promoter. With tamoxifen, the iCreERT2 can enter the nucleus, expressing the effector (TdTomato), which permanently labels activated cells.

### Identifying encoding and retrieval ensembles

**Fig. 3:**
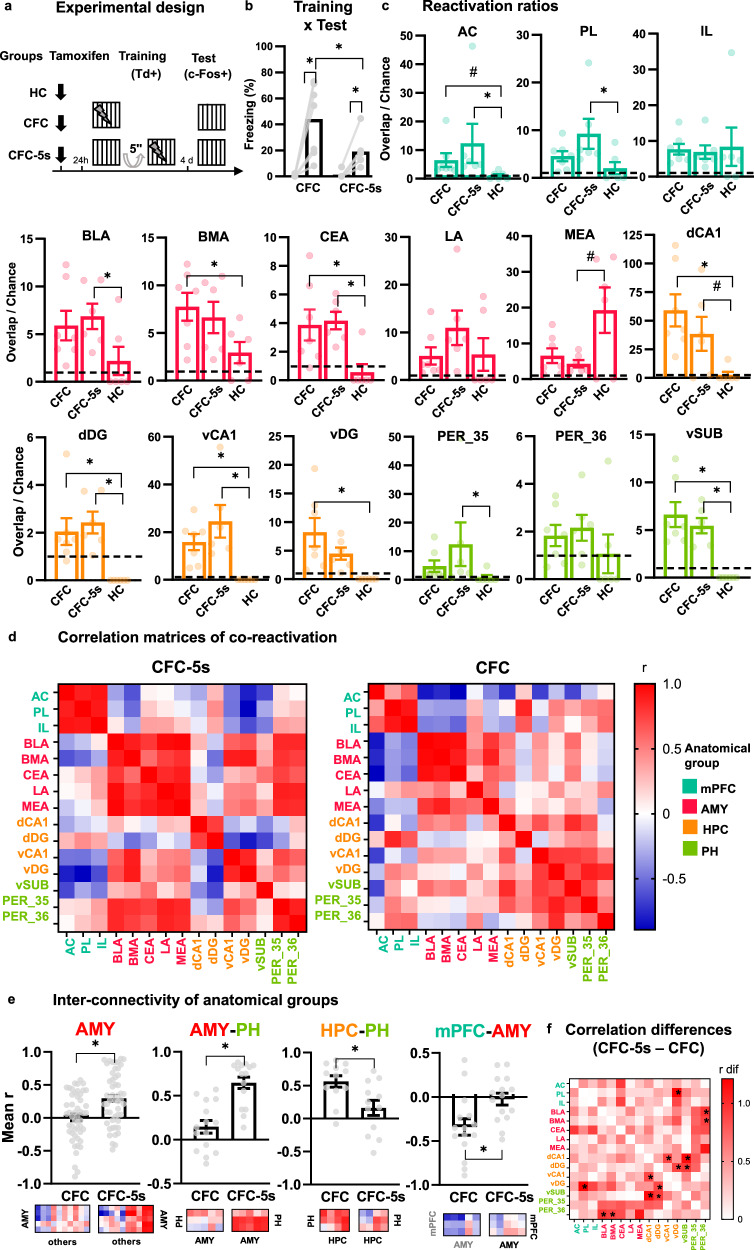
Encoding ensembles reactivated by CFC-5s and CFC retrieval and correlation matrices of their co-reactivation. **a** Experimental design, **b** Mean (±standard error) of the percentage of freezing in the CFC-5s (*n* = 6) and CFC (*n* = 7) groups the training and test sessions. **c** The ratio of observed double-labeled cells (Td- and c-Fos-positive cells standardized by DAPI-positive cells) to chance levels. The dotted lines show overlap/chance ratios equal to 1. * Indicates adjusted *p* < 0.050; **#** indicates a trend toward the statistical significance, with large effect size, and adjusted *p* = 0.058; β = 0.510 in the AC; adjusted *p* = 0.051; β = 1.346 in the MEA, and adjusted *p* = 0.065; β = 0.952 in the dCA1. **d** Pearson’s correlation coefficients of double-labeled cells (Td- and c-Fos-positive cells standardized by DAPI-positive cells) between each pair of regions in the CFC-5s and CFC groups. Colors reflect correlation strengths (scale, right). **e** Mean ( ± standard error) of the mean correlation coefficient (connectivity) between the amygdala nuclei (AMY) and the other regions; between the AMY and parahippocampal area (PH); between the hippocampus (HPC) and PH or between the AMY and medial prefrontal cortex (mPFC). * Indicates adjusted *p* < 0.050. See all comparisons in Supplementary Table 3. **f** Absolute Pearson’s correlation coefficient differences between the CFC-5s and CFC groups. Colors reflect the magnitude of the differences (scale, right). * Indicates significant correlation differences (*p* < 0.050) in the permutation test. See all *p*-values in Supplementary Table 4. Images of labeled cells are represented in Supplementary Figs. 3–6. The strength of the correlation coefficients was not predicted by the magnitude of re-activation in any region (Supplementary Fig. 7). Generalized Linear Models followed by LSD tests controlled for FDR by the Benjamini–Hochberg procedure. Dots show sample distribution. See groups and region names in Table 1.

We mapped the activity of 15 regions following learning and their reactivation following the retrieval of CFC-5s or CFC. We inferred learning-related activity from the TdTomato expression following the training and retrieval-related activity from the c-Fos expression following the test [42–47]. All TRAP2 mice received an intraperitoneal tamoxifen injection (180 mg/kg). We trained them 24 h later in the CFC-5s (*n* = 6) or CFC (*n* = 7) to trap activated cells [42]. We tested CFC-5s and CFC groups four days later in the conditioning chamber for 5 min and euthanized them 90 min later to detect Td- and c-Fos-positive cells. The homecage (HC) group (*n* = 6) remained in the homecage during the training and test sessions. Freezing was measured by FreezeFrame (Fig. 3a).

### Immunofluorescence

Mice were anesthetized with IP injections of Chloral hydrate (400 mg/kg) and perfused with 4% paraformaldehyde. Brains were post-fixed, sectioned in 60-μm coronal sections, and transferred to a blocking solution. In the optogenetic experiments, we incubated the sections with chicken anti-GFP (1:750, H1004, Aves Labs) and then with goat anti-chicken AF-488 (1:500, A11039). In the reactivation experiment, we incubated the sections with mouse anti-RFP (1:1000, 200-301-379, Rockland) and rabbit anti-c-Fos (1:1000, 226003, Synaptic Systems) and then with goat anti-mouse AF-568 (1:500, A11031, Thermofisher) and goat anti-rabbit AF-488 (1:500, A11039). Sections were counterstained with DAPI (1:10000, D9542, Sigma-Aldrich).

### Image analysis

We took six bilateral images, blind to groups, in a laser confocal microscope (Zeiss LSM 710) from the 15 regions shown in Table 1. Regions were chosen based on a previous study investigating activation following CFC-5s learning [37] and memory engrams in CFC [6, 7, 43–56]. Coordinates were consistent among animals (Supplementary Table 1). Automatic scanning obtained adjacent image tiles at 20× (850 ×850 μm) in an optical z-stack of 5 series. Using the Fiji package (ImageJ), we counted DAPI, TdTomato, c-Fos-positive, and double-labeled cells. In the optogenetic experiments, we took six images of the PL to count DAPI- and GFP-positive cells. Only mice with viral infusions restricted to the PL were included.

**Table 1:** Activation of regions following the training or test of the CFC and CFC-5s.

Region	CFC (a)	CFC-5s (b)	HC (c)	Wald	*P*-value
Training [Td+ (% of DAPI+)]					
AC	7.168 ± 1.069^c^	7.026 ± 1.426^c^	4.147 ± 0.928^a,b^	7.147	0.028*
PL	7.414 ± 2.232^c^	5.899 ± 0.517^c^	3.022 ± 0.488^a,b^	12.567	0.002*
IL	4.814 ± 0.860^c^	4.977 ± 0.277^c^	2.581 ± 0.196^a,b^	17.647	0.001*
BLA	10.057 ± 2.595^c^	7.711 ± 2.085^c^	1.475 ± 0.319^a,b^	38.714	0.001*
BMA	9.116 ± 2.613^c^	9.127 ± 2.831^c^	2.850 ± 0.449^a,b^	15.396	0.001*
CEA	15.869 ± 5.426^c^	11.372 ± 2.632^c^	2.540 ± 0.523^a,b^	29.288	0.001*
LA	5.061 ± 1.016^c^	3.665 ± 0.954^c^	1.272 ± 0.120^a,b^	31.011	0.001*
MEA	12.523 ± 3.092^c^	10.806 ± 2.413^c^	2.803 ± 0.352^a,b^	34.513	0.001*
dCA1	1.237 ± 0.342^c^	1.162 ± 0.253^d^	0.572 ± 0.111^a,d^	6.841	0.033*
dDG	5.850 ± 1.187^c^	5.204 ± 0.894^c^	2.975 ± 0.504^a,b^	10.163	0.006*
vCA1	2.408 ± 0.609^c^	1.935 ± 0.157^c^	0.498 ± 0.078^a,b^	50.810	0.001*
vDG	1.902 ± 0.343^c^	2.376 ± 0.351^c^	0.532 ± 0.085^a,b^	44.298	0.001*
vSUB	3.825 ± 0.913^c^	4.566 ± 1.213^c^	0.200 ± 0.028^a,b^	112.91	0.001*
PER_35	8.329 ± 1.752^c^	7.661 ± 2.040^c^	1.598 ± 0.471^a,b^	17.712	0.001*
PER_36	7.774 ± 1.719^c^	5.576 ± 0.949	2.947 ± 0.963^a^	8.458	0.015*
Test [c-Fos + (% of DAPI+)]					
AC	2.000 ± 0.214^c^	1.725 ± 0.426^c^	0.723 ± 0.327^a,b^	10.181	0.006*
PL	3.738 ± 0.792^c^	2.689 ± 0.663^d^	1.015 ± 0.456^a,d^	8.932	0.011*
IL	2.570 ± 0.432	2.329 ± 0.669	1.206 ± 0.486	4.440	0.109
BLA	3.769 ± 0.690^b,c^	2.385 ± 0.285^a,c^	0.793 ± 0.183^a,b^	39.799	0.001*
BMA	4.077 ± 0.746^c^	3.330 ± 0.500^c^	1.457 ± 0.396^a,b^	13.524	0.001*
CEA	2.356 ± 0.263^c^	2.201 ± 0.328^c^	0.558 ± 0.203^a,b^	28.864	0.001*
LA	2.460 ± 0.441^c,d^	1.510 ± 0.239^c,d^	0.582 ± 0.251^a,b^	18.988	0.001*
MEA	4.743 ± 0.924^c^	4.611 ± 0.977^c^	0.697 ± 0.242^a,b^	29.935	0.001*
dCA1	0.431 ± 0.069	0.672 ± 0.419	0.205 ± 0.099	2.041	0.360
dDG	2.743 ± 0.416^c^	3.005 ± 0.649^c^	1.582 ± 0.269^a,b^	9.529	0.009*
vCA1	1.553 ± 0.255^c^	1.323 ± 0.234^c^	0.187 ± 0.084^a,b^	27.644	0.001*
vDG	1.338 ± 0.271^c^	1.318 ± 0.296^c^	0.022 ± 0.022^a,b^	23.209	0.001*
vSUB	3.467 ± 0.524^c^	3.548 ± 0.528^c^	0.073 ± 0.025^a,b^	78.915	0.001*
PER_35	4.756 ± 0.903^c^	3.215 ± 0.400^c^	0.910 ± 0.367^a,b^	20.086	0.001*
PER_36	5.192 ± 0.844^c,d^	3.075 ± 0.336^c,d^	1.438 ± 0.485^a,b^	22.312	0.001*

Encoding and retrieval ensembles induced by training and test in the CFC-5s or CFC. Mean (±standard error) of Td-positive cells or c-Fos-positive cells from the total number of cells (DAPI-positive cells). Generalized Linear Model followed by LSD test controlled by false discovery rate by the Benjamini–Hochberg procedure.

*AC* anterior cingulate cortex, *BLA* basolateral amygdala, *BMA* basomedial amygdala, *CEA* central amygdala, *CFC* contextual fear conditioning, *CFC-5s* CFC with a 5-s interval, *DAPI* 4’,6-diamidino-2-phenylindole, *dCA1* dorsal CA1, *dDG* dorsal dentate gyrus, *IL* infralimbic cortex, *HC* homecage, *LA* lateral amygdala, *MEA* medial amygdala, *PER_35* perirhinal cortex area 35, *PER_36* perirhinal cortex area 36, *PL* prelimbic cortex, *vCA1* ventral CA1, *vDG* ventral dentate gyrus, *vSUB* ventral subiculum.

^a^Indicates adjusted *p* < 0.050 compared to CFC group.

^b^Compared to CFC-5s group.

^c^Compared to HC group.

^d^A trend toward statistical significance, with large effect size, and adjusted *p* = 0.074; β = 0.850 in PL; adjusted *p* = 0.068; β = 0.817 in LA, adjusted *p* = 0.082 β = 0.843 in dCA1; adjusted *p* = 0.073; β = 0.744 in PER_36. See between-group comparisons of DAPI-positive cells in Supplementary Table 1 and Linear Regression Models predicting activity in test by activity in training in Supplementary Table 2.

^*^Indicates p < 0.050.

### Statistical analysis

Data were analyzed by Generalized Linear Models (GZLM) or Generalized Estimating Equations (GEE) using unstructured correlation matrixes for estimations with same-subject observations over time. Estimations were adjusted to Linear, Gamma, or Tweedie probability distributions with the identity link function according to the Akaike Information Criterion score and the Quasi Likelihood under the Independence Model Criterion score in the GZLM and GEE, respectively. For CFC-5s characterization, GEE evaluated the main effect of the group, test session, and their interaction in the freezing time, and GZLM the main effect of the group in the discrimination index (the difference between the freezing time in the threatening and neutral contexts, divided by their sum). For optogenetic experiments, GEE evaluated the main effect of the group, light (ON x OFF periods), and their interaction in the freezing time in mice trained in CFC, CFC-5s, or CFC-5s DIF. GEE also assessed the main effect of the virus, task, and their interaction in the difference of freezing time during the light ON and the light OFF periods in mice trained in CFC-5s and CFC, and GZLM the main effect of group in mice trained in the CFC-5s DIF. For neuronal ensembles, GEE evaluated the main effect of the group, session, and their interaction in the freezing time, and GZLM the main effect of the group in the percentage of Td- and c-Fos-positive cells, and reactivation ratios (Td- and c-Fos-positive cells standardized by DAPI-positive cells) to chance levels, for each region. Chance levels of double-labeled cells were calculated as (Td-positive cells/DAPI-positive cells)*(c-Fos-positive cells/DAPI-positive cells)*100% [47]. *P*-values < 0.050 were considered statistically significant. In these cases, we used the LSD tests (SPSS 23). We conducted the Benjamini–Hochberg procedure in multiple comparisons to control the false discovery rate (FDR). The *p*-values were adjusted globally to maintain an FDR of 5%. All individual *p*-values were put in ascending order and ranked. Adjusted *p*-values were calculated by multiplying the individual *p*-value by the total number of tests, divided by its rank number [57]. We compared effect sizes using standardized regression coefficients (β) [58]. Graphs were generated in GraphPad Prism 8.

### Co-reactivation networks

We used the percentage of reactivated cells (Td- and c-Fos-positive cells standardized by DAPI-positive cells) to generate matrices of Pearson’s correlation between all pairs of regions in the CFC and CFC-5s groups (15 regions, 225 coefficients). We inferred co-reactivation (connections) from the correlation coefficients [59]. We categorized regions into four anatomical groups (mPFC, amygdala, hippocampus, and PH). We computed the mean correlation coefficients (connectivity) between one anatomical group and all the others or between pairs of anatomical groups. GZLM evaluated the main effect of the group in the mean of the correlation coefficients [60]. *P*-values were adjusted by the Benjamini–Hochberg procedure. Permutation tests directly compared each correlation coefficient between groups. They consisted of shuffling the grouping label and re-generating new correlation coefficients. This procedure was repeated 1000 times to generate a null hypothesis distribution. *P*-values were expressed as the frequency that the resampling correlation was higher than the empirical correlation (*p* = resampling difference > empirical difference/1000) [60].

We build networks for each group based on the positive, negative, or both correlations. The latter scaled the relative contribution of each region’s positive and negative correlations [61]. The networks consisted of nodes (regions) connected by edges (connections). GZLM evaluated the effect of the group in topological metrics of global and local efficiency and average cluster coefficient, average and average weighted degree (Supplementary Table 5) [62]. We calculated four centrality measures. The strength (Str) was computed as the average of the correlation coefficients of the node; the eigenvector (Eig) as the sum of the eigenvalues of the neighboring nodes of the node; the betweenness (Bet) as the shortest path between all pairs of nodes passing through that node, and the closeness (Clo) as the average shortest path between the node and others [63, 64]. The upper 25% of nodes in ≥3 centralities were considered hubs, occupying a central network position [64]. Permutation tests directly compared each centrality measure between the CFC-5s and CFC groups for each region. We randomized the group label of each animal without replacement, generated 1000 new networks for each group, and computed their centrality measures, calculating the centrality differences between CFC and CFC-5s networks. The *p*-value was expressed as the frequency of the resampled difference was higher than the observed difference. We also computed each node’s clustering coefficient (CC), a density measure calculated as the number of connected neighbors of a node from the total number of possible connections [65]. We partitioned the networks into communities, subunits of highly interconnected nodes with sparse outsider connections representing functional modules [66, 67]. Nodes were subdivided into clusters of higher within-community than outer-community connections by a modularity optimization algorithm [67]. All graph analyses were performed in R Studio 4 using custom-written routines, which are freely accessible (https://github.com/coelhocao/Brain_Network_analysis) [68] and packages [69–74].

## Results

### Memory specificity depends on the CS-US interval length

We first characterized our temporal association model. We asked whether the context could be fear-associated using matching or mismatching contexts as the CS and US background in an experimental design like tFC. GEE showed a significant effect of group (Wald = 18.632; *p* = 0.001), session (W = 52.774; *p* = 0.001), and their interaction (W = 27.448; *p* = 0.001) in the freezing time. All groups exhibited significantly higher freezing times in their threatening than neutral contexts (CFC: *p* = 0.001; β = 0.892; CFC-5s: *p* = 0.001; β = 0.582; CFC-5s DIF 1: *p* = 0.001; β = 1.222; CFC DIF 2: *p* = 0.025; β = 1.172), indicating that freezing was context-specific in all temporal association designs (Fig. 1c). The CFC (*p* = 0.001; β = 0.310) and CFC-5s DIF 1 (*p* = 0.001; β = 1.621) groups also had higher freezing times than the CFC-5s group in its threatening context. The CFC-5s DIF 1 group had higher contextual discrimination. GZLM showed a significant effect of group (GZLM W = 14.544; *p* = 0.002) in the discrimination index. The CFC-5s DIF 1 had a higher discrimination index than CFC (*p* = 0.001; β = 1.367) and CFC-5s (*p* = 0.001; β = 1.294) groups and similar to the CFC-5s DIF 2 group (*p* = 0.226; β = 0.953; Fig. 1e).

Next, we asked whether the increase in the CS-US interval length would decrease memory retention, a common feature of other temporal associations. GEE showed a significant effect of group (W = 35.398; *p* = 0.001), session (W = 16.938; *p* = 0.001) and their interaction (W = 9.490; *p* = 0.050) in the freezing time. The 15 s (*p* = 0.003; β = 1.168), 30 s (*p* = 0.015; β = 1.122), 1 min (*p* = 0.011; β = 0.732), and 10 min (*p* = 0.001; β = 0.763) groups exhibited significantly higher freezing times in the threatening than in the neutral context. The 15 s (*p* = 0.001; β = 1.775), 30 s (*p* = 0.014; β = 1.214), and 10 min (*p* = 0.039; β = 1.012) groups also had higher freezing times in the threatening context than the 60 min group, and the 15 s group than the 1 min (*p* = 0.001; β = 1.127) and the 10 min groups (*p* = 0.048; β = 0.763), indicating a decrease in the CR with the increase of the interval (Fig. 1d). Contextual discrimination decreased at 60-minute intervals. GZLM showed a significant group effect in the discrimination index (W = 13.636; *p* = 0.009). The 15 s (*p* = 0.015; β = 1.062), 30 s (*p* = 0.004; β = 1.227), 1 min (*p* = 0.017; β = 0.986), and 10 min (*p* = 0.001; β = 1.507) groups had a significantly higher discrimination index than the 60 min group (Fig. 1f). The results suggest that the context is subject to tFC, and memory was context-specific in intervals of up to 10 min and decreased with longer intervals.

### Reactivating or silencing PL ensembles recovers or impairs the CFC-5s memory

We evaluated whether the reactivation of PL ensembles encoding CFC-5s memory induces memory retrieval. We tagged PL ensembles encoding CFC-5S training, reactivated them in a neutral context, and observed their effects on freezing responses (Fig. 2d). GEE showed a significant effect of the light (W = 16.501; *p* = 0.001) and the interaction between the group and light (W = 15.067; *p* = 0.001). The ChR2 group exhibited higher freezing during light ON than OFF (*p* = 0.001; β = 1.736) and than the GFP group during light ON (*p* = 0.006; β = 1.320) or OFF (*p* = 0.002; β = 1.696). In contrast, the reactivation of PL ensembles did not increase freezing in CFC-trained mice (Fig. 2e). GEE showed a significant light effect (W = 27.095; *p* = 0.001), with higher freezing during light ON, independent of the group. We used the freezing difference (ON–OFF) to directly compare the CFC-5s and CFC groups (Fig. 2f). GEE showed a significant effect of the virus (W = 5.706; *p* = 0.017), task (W = 6.170; *p* = 0.013), and their interaction (W = 8.416; *p* = 0.004) in the freezing difference. The freezing difference was higher in the ChR2/CFC-5s group than in all other groups (GFP/CFC-5s: *p* = 0.005; β = 1.876; GFP/CFC: *p* = 0.005; β = 2.118; ChR2/CFC: *p* = 0.001; β = 1.517).

We next evaluated whether PL ensembles encoding CFC-5s memory are required for memory retrieval, silencing them in the threatening context to observe its effects on freezing responses (Fig. 2g). GEE showed a significant effect of light (W = 30.574; *p* = 0.001) and group and light interaction (W = 12.839; *p* = 0.001). Freezing was lower in the NpACY/CFC-5s group during light ON than OFF (*p* = 0.001; β = 1.820) and than the GFP/CFC-5s group during light ON (*p* = 0.010; β = 0.694) or OFF (*p* = 0.001; β = 1.431). Inhibition of encoding ensembles in PL did not decrease freezing in CFC-trained mice (Fig. 2h). GEE did not show a significant effect of the group (W = 0.034; *p* = 0.854), the light (W = 1.382; *p* = 0.240), or their interaction (W = 3.109; *p* = 0.078) in the freezing time, but a significant effect of the task (W = 10.690; *p* = 0.001) and the task and virus interaction (W = 13.578; *p* = 0.001) in the freezing difference, which was lower in the NpACY/CFC-5s group than all the other ones (GFP/CFC-5s: *p* = 0.001; β = 1.423; GFP/CFC: *p* = 0.001; β = 1.317; NpACY/CFC: *p* = 0.001; β = 1.779; Fig. 2i).

The same result pattern was observed using another temporal association, the CFC-5s DIF task (Fig. 2j–k). Freezing promoted by the opto-reactivation (artificially evoked) and exposure to the threatening context (CS produced) were similar and higher than in the neutral context or during the silencing period. GEE showed a significant virus and light interaction (W = 33.370; *p* = 0.001) in the freezing time, which was higher in the ChR2 group during light ON than OFF (*p* = 0.001; β = 1.486) and than in the NpACY group during light ON (*p* = 0.003; β = 1.139). Freezing was lower in the NpACY group during the light ON than OFF (*p* = 0.001; β = 1.147). The ChR2 group had a higher freezing difference than the NpACY group (GZLM W = 33.151; *p* = 0.001; β = 1.633). The viral constructs have previously been shown to increase or decrease c-Fos after opto-reactivation or opto-inhibition [75]. These results suggest that PL encoding ensembles are necessary, and their reactivation promotes retrieval-like behavior in temporal associations.

### CFC-5s had co-localized encoding and retrieval ensembles in the PL, BLA, and PER_35

Activation of cells following both the training and test sessions was used to infer neuronal ensembles supporting temporal associations (Fig. 3a). Reactivation could occur randomly in the same cells activated in training. Thus, we used the ratio of observed reactivation to chance levels [43–47, 76]. GEE showed a significant effect of the group (W = 4.514; *p* = 0.034), session (W = 35.579; *p* = 0.001) and their interaction (W = 66.254; *p* = 0.012) in the freezing time. Freezing to the threatening context was higher in the test than in training in CFC-5s (*p* = 0.013; β = 0.635) and CFC (*p* = 0.001; β = 1.891) groups, which had higher freezing than CFC-5s group in the test (*p* = 0.021; β = 0.711; Fig. 3b).

GZLM controlled for FDR showed a significant group effect on the reactivation ratio in the AC (W = 12.558; *p* = 0.002), BLA (W = 6.380; =0.041), BMA (W = 7.298; =0.026), CEA (W = 13.200; *p* = 0.001), dCA1 (W = 12.346; =0.002), dDG (W = 20.171; =0.001), MEA (W = 17.946; =0.001), PER_35 (W = 8.681; *p* = 0.013), PL (W = 6.508; *p* = 0.039), vCA1 (W = 18.741; *p* = 0.001), vDG (W = 14.233; *p* = 0.001), and vSUB (W = 32.603; =0.001), but not in the IL (W = 0.147; *p* = 0.929), LA (W = 1.541; *p* = 0.463), and PER_36 (W = 1.961; *p* = 0.375). Both the CFC-5s and CFC groups had a higher reactivation ratio than the HC group in the CEA (CFC-5s adjusted *p* = 0.006; β = 1.401; CFC adjusted *p* = 0.011; β = 1.282), dDG (CFC-5s adjusted *p* = 0.001; β = 1.622; CFC adjusted *p* = 0.001; β = 1.366), vCA1 (CFC-5s adjusted *p* = 0.001; β = 1.700; CFC adjusted *p* = 0.020; β = 1.098), and vSUB (CFC-5s adjusted *p* = 0.001; β = 1.471; CFC adjusted *p* = 0.001; β = 1.786); the CFC also in BMA (adjusted *p* = 0.034; β = 1.204), dCA1 (adjusted *p* = 0.025; β = 1.498), and vDG (adjusted *p* = 0.001; β = 1.545), and the CFC-5s also in the AC (adjusted *p* = 0.023; β = 1.062), PL (adjusted *p* = 0.050; β = 1.304), BLA (adjusted *p* = 0.050; β = 1.158), and PER_35 (adjusted *p* = 0.050; β = 1.012; Fig. 3c). The CFC-5s group had a trend toward a higher reactivation ratio, with a large effect size in the dCA1 (adjusted *p* = 0.065; β = 0.952); the CFC group in the AC (*p* = 0.058; β = 0.510), and the HC in the MEA (adjusted *p* = 0.051; β = 1.346). Therefore, the CFC and CFC-5s groups were not directly different in any region, but only the CFC group increased the reactivation ratio in the BMA and vDG compared to the HC, and the CFC-5s group in the PL, BLA, and PER_35, suggesting an indirect difference between them when compared to the HC group.

The results of GZLM controlled for FDR evaluating the effect of the group in Td- and c-Fos-positive cells are fully reported in Table 1. They showed that CFC-5s and CFC have encoding and retrieval ensembles in similar regions. CFC learning activated all investigated areas, as well as CFC retrieval, except the IL and dCA1. In turn, CFC-5s learning did not activate the PER_36, and CFC retrieval activated the BLA more than CFC-5s retrieval, in line with the higher freezing time of CFC than CFC-5s group in the test session.

### CFC-5s strengthened amygdala-PH connectivity

We investigated whether the interval changes connections between regions or connectivity between anatomical groups (Fig. 3d). Given that CFC and CFC-5s are forms of context-US associations and shared neuronal ensembles in the same regions, differences could be reflected in how these discrete regions are functionally connected and organized at the network level. GZLM controlled for FDR showed that the CFC-5s group had higher mean correlation coefficients (connectivity) of reactivated cells (Td- and c-Fos-positive cells standardized by DAPI-positive cells) than the CFC group between the amygdala and other regions (GZLM W = 14.271; adjusted *p* = 0.001; β = 0.703), the amygdala and mPFC (GZLM W = 8.120; adjusted *p* = 0.004; β = 0.908), and the amygdala and PH (GZLM W = 28.641; adjusted *p* = 0.001; β = 1.374). The CFC group had higher connectivity between the hippocampus and PH (GZLM W = 8.366; adjusted *p* = 0.004; β = 0.995; Fig. 3e). We then directly compared the groups’ connection (correlation coefficients) (Fig. 3f). Permutation tests showed higher PER_36-BLA (*p* = 0.017) and PER_36-BMA (*p* = 0.050) positive connections in the CFC-5s than the CFC group, and higher vDG-PL (*p* = 0.001), vDG-dDG (*p* = 0.033), dCA1-vCA1 (*p* = 0.050), vSUB-dCA1 (*p* = 0.001), and vSUB-dDG (*p* = 0.033) positive connections in the CFC than the CFC-5s group, suggesting that temporal associations increase amygdala-PH connectivity and reduce intra-hippocampal or hippocampus-PH connectivity.

### CFC-5s network had hubs and higher centralities in the amygdala and PH

**Fig. 4:**
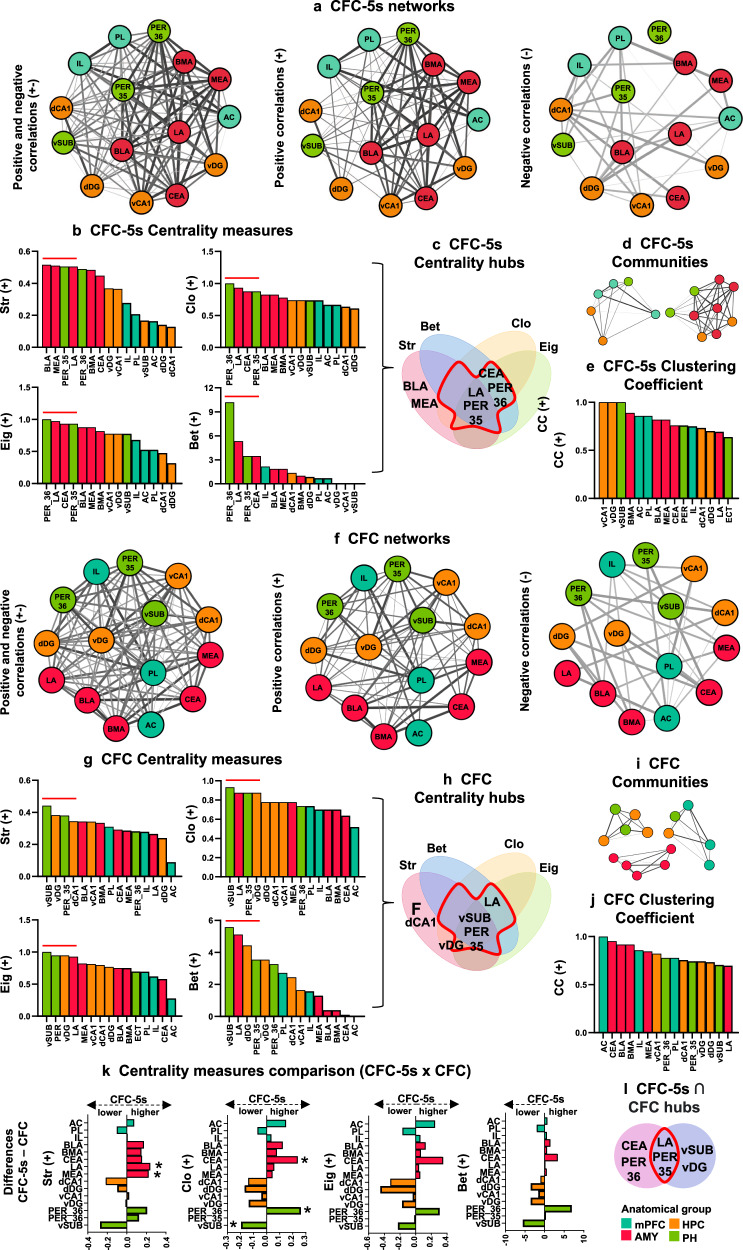
Co-reactivation networks of CFC-5s and CFC. **a**, **f** Positive and negative, positive, and negative weight networks of CFC-5s and CFC. Nodes represent regions. Colors indicate the node’s anatomical group (scale, bottom right). Edges between nodes represent correlation coefficients. Edge thickness and gray scale are proportional to the correlation strength. See network overviews in Supplementary Table 5 and edge analysis in Supplementary Fig. 8. **b**, **g** Regions ranked in a decrescent order of the centrality measures of strength (Str), closeness (Clo), eigenvector (Eig), and betweenness (Bet) in positive weight networks. **c**, **h** The upper 25% of regions in three or more centrality measures were considered hubs (inside the red perimeter). **d**, **i** Positive weight networks are subdivided into communities. **e**, **j** Regions ranked in a decrescent of the Clustering Coefficient (CC). **k** Centrality differences between CFC-5s and CFC groups. * Indicates significant centrality differences (*p* < 0.050) in the permutation test. See the *p*-values of all permutation tests in Supplementary Table 6. **l** Hubs of CFC-5s and CFC networks were intersected to identify stable hubs in fear conditioning (inside the red perimeter). See network analyses of negative weight networks in Supplementary Fig. 9. To verify whether hubs impacted the network topology more than low centrality regions, we compared the efficiency of CFC-5s or CFC networks without hubs or non-hubs nodes in Supplementary Table 7. See groups and region names in Table 1.

We questioned whether temporal associations change the organization and importance of regions in networks. Using the percentage of reactivated cells, we build co-reactivation networks for CFC and CFC-5s groups (Fig. 4a, f). The CFC-5s and CFC had one community with mPFC, DH, and PER_36; the CFC one with amygdala and another with HPC and PER_35/vSUB, which formed a single community in CFC-5s—(Fig. 4d, i). Thus, the amygdala, VH, and PER_35/vSUB were clustered in CFC-5s, indicating a similar functionality.

The PH and hippocampus had the highest centralities in the CFC positive weight network, and the PH and amygdala in the CFC-5s positive weight network (Fig. 4b, g). The CFC-5s and CFC networks had the PER_35 and LA as hubs; the CFC also the vSUB and vDG, and the CFC-5s the CEA and PER_36 (Fig. 4c, h, l). We then directly compared the node’s centralities between groups in positive weight networks (Fig. 4k). Permutation tests showed that the LA (*p* = 0.050) and MEA (=0.001) had a higher Str and the CEA (*p* = 0.050) and PER_36 (*p* = 0.050) a higher Clo in the CFC-5s than in the CFC positive weight network, and the vSUB a higher Clo in the CFC network than in the CFC-5s network (*p* = 0.001). Thus, the CEA, LA, and PER_36 were hubs with higher centralities in CFC-5s than the CFC network and the vSUB in the CFC network. Sex was not a significant factor in any statistical analyses.

Connectivity and network analyses consistently indicated that the amygdala and PH (PER_36) are highly interconnected and more central in the CFC-5s network, and the PH (vSUB) and hippocampus (vDG) in the CFC network, in line with the higher reactivation of PER_35 by CFC-5s and dCA1/vDG by CFC.

## Discussion

We investigated neuronal ensembles of temporal associations, which link transient memories of past stimuli with subsequent ones. The reactivation of PL encoding ensembles accompanied, facilitated, and was necessary for memory retrieval of temporal associations, which had increased amygdala and PER centralities and strengthened amygdala-PH connectivity in networks.

Accordingly, previous studies have shown that pre-training PL inactivation impairs the encoding of temporal associations [77–80]. The PL also had post-training learning-related changes [36, 37, 77]. Studies targeting the mPFC have investigated it in retrieving temporal associations, showing that post-training or pre-test inhibition impaired consolidation or disrupted retrieval [77, 81, 82]. Present findings showed PL participation in memory retrieval and a causal relationship between learning-related PL activity and freezing, suggesting that the same PL neurons participating in learning are required for retrieving temporal associations. Future studies can dissect which cell subtypes/projections form neuronal ensembles in the PL.

CFC did not require activation of PL encoding ensembles for memory retrieval, suggesting they are related to temporal associations and not to CFC, contextual, or non-associative learning. Reactivating or inhibiting PL retrieval ensembles, but not encoding ensembles, facilitated and impaired the remote retrieval of CFC, suggesting that PL ensembles reorganize over time, being distinct from those activated by learning [14, 15]. Our results extend these findings to the recent retrieval of CFC. Only studies examining mPFC encoding ensembles have investigated their participation in recent memories. They showed that opto-reactivating mPFC encoding ensembles induced, but opto-inhibiting them did not prevent recent retrieval. In turn, opto-reactivating or opto-inhibiting mPFC encoding ensembles promoted or impaired remote retrieval, which are results different from those manipulating PL ensembles [14–17]. The effects observed by mPFC studies may be due to other mPFC subdivisions or require all of them.

The AC is required for the recent and remote consolidation of the CFC, having post-training plasticity necessary for memory retrieval [83–88], while pre-test AC inhibition spares recent retrieval, consistent with results from opto-stimulation of mPFC encoding ensembles [16, 89–91]. Opto-reactivation of mPFC encoding ensembles could reactivate other regions to support recent retrieval, while opto-reactivation of PL alone may not be sufficient.

The PL was not a hub nor had high centralities in the co-reactivation networks of CFC-5s, corroborating previous results from CFC-5s learning networks [37]. Claustrum-PL and insula-PL projections were required for learning and recent retrieval of CFC [28]. Including claustrum and insula may have increased PL connectivity, or other activity markers could better detect PL co-reactivations. Moreover, the CFC strengthened negative amygdala-mPFC connectivity. Like a human CFC network, the AC was negatively correlated with the BLA and CEA [92].

CFC-5s reactivation increased amygdala connectivity and centrality, like the CFC-5s learning network [37]. Because there are no salient stimuli during the US, past or broader stimuli could be considered predictive and be converged into the amygdala via multiple pathways to be fear-associated, increasing its connectivity. Other stimuli may have reduced hippocampal participation or preferentially engaged regions supporting contextual learning and persistent activity, such as the PER [93–97].

Indeed, the CFC-5s increased amygdala-PH connectivity. The LA/BLA and PER have reciprocal projections and endogenous persistent-firing neurons and are required for tFC [98–100]. The PER also has a binding mechanism, which unitizes stimuli [93, 94]. Moreover, the PER, amygdala, and VH were functional modules in the CFC-5s network. The PER could maintain transient CS memories conveyed by/to the VH through persistent firing or unitizing mechanisms and associate it with the US in the amygdala. Future studies can verify the requirement of PER-amygdala projections in temporal associations and whether the hubs identified are also required for CFC-5s. Silico deletion of hubs impaired the efficiency of a fear memory network and their inactivation memory consolidation, suggesting that hub identification can predict biological relevance [101].

Our results align with studies showing that the amygdala and DG/CA1 have encoding ensembles reactivated by CFC retrieval [13, 43–51, 76]. Their reactivation or inhibition promoted or impaired memory retrieval of fear conditioning [7, 45, 48, 52–56, 76]. We expanded their participation to temporal association memories. Their reactivation could be due to reconsolidation or extinction. However, nonoverlapping neurons form encoding and extinction ensembles [56, 76, 102]. Moreover, CFC networks had higher hippocampal and PH interconnectivity and centralities. CA1 and DG ensembles were related to contextual learning and pattern separation [49]. The SUB is required for CFC and has plasticity-related changes correlated with CR [103–105].

Differences between CFC-5s and CFC ensembles were detected at the connectivity and network levels and in the necessity/sufficiency of regions rather than in their activation/reactivation. Thus, CFC-5s and CFC may involve similar areas connected differently or more crucial in the CFC-5s than in the CFC. The same regions could participate in both learnings but performing additional functions in CFC-5s, such as CS short-term memory and attention, which could require functional connections to other areas, making differences less easily detected by the magnitude of activation than connectivity. Looking at the activation only during the 5-s interval may reveal differences at discrete regions. Alternatively, increasing the sample could improve our power to detect more direct between-group differences in the activation/reactivation of regions.

Increasing the interval decreased the memory specificity in CFC-5s, like decreased performance in working memory tasks [106]. Longer intervals could increase distraction opportunities, impairing the transient memory. The CFC-5s indicated that past environments can be associated with future aversive events. Since space is ubiquitous, contexts temporally linked to traumatic experiences can be a new target for exposure therapies to reduce maladaptive fear.

PL encoding ensembles are required for retrieving temporal associations, which has increased amygdala and PER interconnectivity and centralities. We characterized encoding and retrieval ensembles of temporal associations and their co-reactivation network for the first time, which can improve our understanding of dysfunctions in temporal associations that accompany aging, neuropsychiatric, or neurodegenerative conditions.

### Supplementary information

Supplemental Material

## References

[1] Raybuck JD, Lattal KM (2014). Bridging the Interval: Theory and Neurobiology of Trace Conditioning. Behav Processes.

[2] Pilkiw M, Takehara-Nishiuchi K (2018). Neural representations of time-linked memory. Neurobiol Learn Memory.

[3] McEchron MD, Cheng AY, Gilmartin MR (2004). Trace fear conditioning is reduced in the aging rat. Neurobiol Learn Mem.

[4] Ohno M, Chang L, Tseng W, Oakley H, Citron M, Klein WL (2006). Temporal memory deficits in Alzheimer’s mouse models: rescue by genetic deletion of BACE1. Eur J Neurosci.

[5] Koh MT, Shao Y, Sherwood A, Smith DR (2016). Impaired hippocampal-dependent memory and reduced parvalbumin-positive interneurons in a ketamine mouse model of schizophrenia. Schizophr Res.

[6] Josselyn SA, Tonegawa S (2020). Memory Engrams: Recalling the Past and Imagining the Future. Science.

[7] Roy DS, Park Y, Ogawa SK, Cho JH, Choz H, Kamensky L (2022). Brain-wide mapping reveals that engrams for a single memory are distributed across multiple brain regions. Nat Commun.

[8] Semon RW (1923). Mnemic Psychology.

[9] Pavlov I (1927). Conditioned reflexes: An investigation of the physiological activity of the cerebral cortex.

[10] Baeg EH, Kim YB, Jang J, Kim HT, Mook-Jung I, Jung MW (2001). Fast Spiking and Regular Spiking Neural Correlates of Fear Conditioning in the Medial Prefrontal Cortex of the Rat. Cerebral Cortex.

[11] Gilmartin MR, McEchron MD (2005). Single Neurons in the Medial Prefrontal Cortex of the Rat Exhibit Tonic and Phasic Coding During Trace Fear Conditioning. Behav Neurosci.

[12] Gilmartin MR, Miyawaki H, Helmstetter FJ, Diba K (2013). Prefrontal Activity Links Nonoverlapping Events in Memory. J Neurosci.

[13] Zelikowsky M, Hersman S, Chawla MK, Barnes CA, Fanselow MS (2014). Neuronal Ensembles in Amygdala, Hippocampus, and Prefrontal Cortex Track Differential Components of Contextual Fear. J Neurosci.

[14] DeNardo LA, Liu CD, Allen WE, Adams EL, Friedmann D, Fu L (2019). Temporal Evolution of Cortical Ensembles Promoting Remote Memory Retrieval. Nat Neurosci.

[15] Giannotti G, Jasper A, Heinsbroek A, Yue J, Deisseroth K, Peters J (2019). Prefrontal Cortex Neuronal Ensembles Encoding Fear Drive Fear Expression During Long-Term Memory Retrieval. Sci Rep.

[16] Kitamura T, Ogawa SK, Roy DS, Okuyama T, Morrissey MD, Smith LM (2017). Engrams and Circuits Crucial for Systems Consolidation of a Memory. Science.

[17] Matos MR, Visser E, Kramvis I, van der Loo RJ, Gebuis T, Zalm R (2019). Memory strength gates the involvement of a CREB-dependent cortical fear engram in remote memory. Nat Commun.

[18] Corcoran KA, Quirk GJ (2007). Activity in Prelimbic Cortex Is Necessary for the Expression of Learned, but Not Innate, Fears. J Neurosci.

[19] Xu W, Südhof TC (2013). A Neural Circuit for Memory Specificity and Generalization. Science.

[20] Santos TB, Kramer-Soares JC, Favaro VM, Oliveira MGM (2017). Involvement of the Prelimbic Cortex in Contextual Fear Conditioning with Temporal and Spatial Discontinuity. Neurobiol Learn Memory.

[21] Heroux NA, Horgan CJ, Stanton ME (2021). Prefrontal Nmda-Receptor Antagonism Disrupts Encoding or Consolidation but Not Retrieval of Incidental Context Learning. Behav Brain Res.

[22] Choi DC, Maguschak KA, Ye K, Jang SW, Myers KM, Ressler KJ (2010). Prelimbic Cortical Bdnf Is Required for Memory of Learned Fear but Not Extinction or Innate Fear. Proc Natl Acad Sci USA.

[23] Gilmartin MR, Kwapis JL, McEchron MD (2012). Trace and Contextual Fear Conditioning Are Impaired Following Unilateral Microinjection of Muscimol in the Ventral Hippocampus or Amygdala, but Not the Medial Prefrontal Cortex. Neurobiol Learn Memory.

[24] Mukherjee A, Caroni P (2018). Infralimbic Cortex Is Required for Learning Alternatives to Prelimbic Promoted Associations through Reciprocal Connectivity. NatCommun.

[25] Asok A, Schreiber WB, Jablonski SA, Rosen JB, Stanton ME (2013). Egr-1 Increases in the Prefrontal Cortex Following Training in the Context Preexposure Facilitation Effect (CPFE) Paradigm. Neurobiol Learn Memory.

[26] Rizzo V, Touzani K, Raveendra BL, Swarnkar S, Lora J, Kadakkuzha BM (2017). Encoding of Contextual Fear Memory Requires De Novo Proteins in the Prelimbic Cortex. Biol Psychiatr.

[27] Cummings KA, Clem RL (2020). Prefrontal Somatostatin Interneurons Encode Fear Memory. Nat Neurosci.

[28] Dixsaut L, Gräff J (2022). Brain-wide screen of prelimbic cortex inputs reveals a functional shift during early fear memory consolidation. eLife.

[29] Burgos-Robles A, Vidal-Gonzalez I, Quirk GJ (2009). Sustained Conditioned Responses in Prelimbic Prefrontal Neurons Are Correlated with Fear Expression and Extinction Failure. J Neurosci.

[30] Laurent V, Westbrook RF. Inactivation of the Infralimbic but Not the Prelimbic Cortex Impairs Consolidation and Retrieval of Fear Extinction. Learn Memory. 2009;16:520–9. 10.1101/lm.1474609 19706835

[31] Sierra-Mercado D, Padilla-Coreano N, Quirk GJ (2011). Dissociable Roles of Prelimbic and Infralimbic Cortices, Ventral Hippocampus, and Basolateral Amygdala in the Expression and Extinction of Conditioned Fear. Neuropsychopharmacology.

[32] Stevenson CW (2011). Role of Amygdala-Prefrontal Cortex Circuitry in Regulating the Expression of Contextual Fear Memory. Neurobiol Learn Memory.

[33] Courtin J, Chaudun F, Rozeske RR, Karalis N, Gonzalez-Campo C, Wurtz H (2014). Prefrontal Parvalbumin Interneurons Shape Neuronal Activity to Drive Fear Expression. Nature.

[34] Nambu MF, Lin YJ, Reuschenbach J, Tanaka KZ (2022). What does engram encode? Heterogeneous memory engrams for different aspects of experience. Curr Opin Neurobiol.

[35] Santos TB, Wallau AE, Kramer-Soares JC, Oliveira MGM (2020). Functional interaction of ventral hippocampal CA1 region and prelimbic cortex contributes to the encoding of contextual fear association of stimuli separated in time. Neurobiol Learn Memory.

[36] Santos TB, Kramer-Soares JC, Oliveira MGM (2023). Contextual fear conditioning with a time interval induces CREB phosphorylation in the dorsal hippocampus and amygdala nuclei that depend on prelimbic cortex activity. Hippocampus.

[37] Santos TB, Kramer-Soares JC, de Oliveira Coelho CA, Oliveira MGM (2023). Functional network of contextual and temporal memory has increased amygdala centrality and connectivity with the retrosplenial cortex, thalamus, and hippocampus. Sci Rep.

[38] Faul F, Lang A, Buchner AG. *Power 3: A flexible statistical power analysis program for the social, behavioral, and biomedical sciences. Behav Res Methods. 2007;39:175–91. 10.3758/bf03193146 17695343

[39] Sørensen AT, Cooper YA, Baratta MV, Weng F, Zhang Y, Ramamoorthi K (2016). A Robust Activity Marking System for Exploring Active Neuronal Ensembles. eLife.

[40] Franklin KBJ, Paxinos G (2007). The mouse brain in stereotaxic coordinates.

[41] Allen WE, DeNardo LA, Chen MZ, Liu CD, Loh KM, Fenno LE (2017). Thirst-associated preoptic neurons encode an aversive motivational drive. Science.

[42] Guenthner CJ, Miyamichi K, Yang HH, Heller HC, Luo L (2013). Permanent Genetic Access to Transiently Active Neurons Via Trap: Targeted Recombination in Active Populations. Neuron.

[43] Ramirez S, Liu X, Lin PA, Suh J, Pignatelli M, Redondo RL (2013). Creating a False Memory in the hippocampus. Science.

[44] Tayler KK, Tanaka KZ, Reijmers LG, Wiltgen BJ (2013). Re-activation of Neural Ensembles During the Retrieval of Recent and Remote Memory. Curr Biol.

[45] Tanaka KZ, Pevzner A, Hamidi AB, Nakazawa Y, Graham J, Wiltgen BJ (2014). Cortical Representations Are Reinstated by the Hippocampus During Memory Retrieval. Neuron.

[46] Nakazawa Y, Pevzner A, Tanaka KZ, Wiltgen BJ (2016). Memory Retrieval Along the Proximodistal Axis of CA1. Hippocampus.

[47] Reijmers LG, Perkins BL, Matsuo N, Mayford M (2007). Localization of a Stable Neural Correlate of Associative Memory. Science.

[48] Ghandour K, Ohkawa N, Fung CCA, Asai H, Saitoh Y, Takekawa T (2019). Orchestrated Ensemble Activities Constitute a Hippocampal Memory Engram. Nat Commun.

[49] Deng W, Mayford M, Gage FH (2013). Selection of Distinct Populations of Dentate Granule Cells in Response to Inputs as a Mechanism for Pattern Separation in Mice. eLife.

[50] Trouche S, Sasaki JM, Tu T, Reijmers LG (2013). Fear Extinction Causes Target-Specific Remodeling of Perisomatic Inhibitory Synapses. Neuron.

[51] Denny CA, Kheirbek MA, Alba EL, Tanaka KF, Brachman RA, Laughman KB (2014). Hippocampal Memory Traces Are Differentially Modulated by Experience, Time, and Adult Neurogenesis. Neuron.

[52] Liu X, Ramirez S, Pang PT, Puryear CB, Govindarajan A, Deisseroth K (2012). Optogenetic Stimulation of a Hippocampal Engram Activates Fear Memory Recall. Nature.

[53] Ryan TJ, Roy D, Pignatelli M, Arons A, Tonegawa S (2015). Engram Cells Retain Memory under Retrograde Amnesia. Science.

[54] Park S, Kramer EE, Mercaldo V, Rashid AJ, Insel N, Frankland PW (2016). Neuronal Allocation to a Hippocampal Engram. Neuropsychopharmacology.

[55] Roy DS, Muralidhar S, Smith LM, Tonegawa S (2017). Silent Memory Engrams as the Basis for Retrograde Amnesia. Proc Natl Acad Sci USA.

[56] Zhang X, Kim J, Tonegawa S (2020). Amygdala Reward Neurons Form and Store Fear Extinction Memory. Neuron.

[57] Benjamini Y, Hochberg Y (1995). Controlling the False Discovery Rate: A Practical and Powerful Approach to Multiple Testing. R Stat Soc.

[58] Cohen J (1992). A Power primer. Psychol Bull.

[59] Park HJ, Friston K (2013). Structural and functional brain networks: from connections to cognition. Science.

[60] Wheeler AL, Teixeira CM, Wang AH, Xiong X, Kovacevic N, Lerch JP (2013). Identification of a Functional Connectome for Long-Term Fear Memory in Mice. PLOS Comput Biol.

[61] Rubinov M, Sporns O (2010). Complex network measures of brain connectivity uses and interpretations. Neuroimage.

[62] Latora V, Marchiori M (2011). Efficient behavior of small-world networks. Phys Rev Lett.

[63] Ruhnau B (2000). Eigenvector-centrality - a node-centrality?. Soc Netw.

[64] Van den Heuvel MP, Sporns O (2011). Rich-club organization of the human connectome. J Neurosci.

[65] Watts DJ, Strogatz SH (1998). Collective dynamics of ‘small-world’ networks. Nature.

[66] Guimerà R, Amaral LA. Cartography of complex networks: modules and universal roles. J Stat Mech. 2005;P02001:nihpa35573. 10.1088/1742-5468/2005/02/P02001 PMC2151742 18159217

[67] Blondel VD, Guillaume JL, Lambiotte R, Lefebvre E. Fast unfolding of communities in large networks. arxiv. 2008. https://arxiv.org/abs/0803.0476.

[68] Coelho CAO, Ferreira TL, Kramer-Soares JC, Sato JR, Oliveira MGM (2018). Network Supporting Contextual Fear Learning after Dorsal Hippocampal Damage Has Increased Dependence on Retrosplenial Cortex. PLOS Comput Biol.

[69] Csardi G, Nepusz T. The igraph software package for complex network research. InterJ Complex Syst. 2006:1695. https://cran.r-project.org/web/packages/igraph/citation.html.

[70] Bates D, Machler M. R package ‘Matrix’: Sparse and Dense Matrix Classes and Methods. (Version 1.5-4). 2015. https://cran.r-project.org/web/packages/Matrix/Matrix.pdf.

[71] Sakar D. Lattice: Multivariate Data Visualization with R. New York: Springer; 2008.

[72] Wei T, Simko V. R package ‘corrplot’: Visualization of a Correlation Matrix. (Version 0.92). 2021. https://cran.r-project.org/web/packages/corrplot/corrplot.pdf.

[73] Fox J, Weisberg S (2011). An R Companion to Applied Regression.

[74] Chen H, Boutros PC (2011). VennDiagram: a package for the generation of highly-customizable Venn and Euler diagrams in R. BMC Bioinformatics.

[75] Lau JMH, Rashid AJ, Jacob AD, Frankland PW, Schacter DL, Josselyn SA (2020). The Role of Neuronal Excitability, Allocation to an Engram and Memory Linking in the Behavioral Generation of a False Memory in Mice. Neurobiol Learn Memory.

[76] Lacagnina AF, Brockway ET, Chelsea R, Crovetti FS, McCarty MJ, Sattler KP (2019). Distinct Hippocampal Engrams Control Extinction and Relapse of Fear Memory. Nat Neurosci.

[77] Runyan JD, Dash PK (2004). Intra-Medial Prefrontal Administration of Sch-23390 Attenuates Erk Phosphorylation and Long-Term Memory for Trace Fear Conditioning in Rats. Neurobiol Learn Memory.

[78] Gilmartin MR, Helmstetter FJ (2010). Trace and Contextual Fear Conditioning Require Neural Activity and Nmda Receptor-Dependent Transmission in the Medial Prefrontal Cortex. Learn Memory.

[79] Guimarãis M, Gregório A, Cruz A, Guyon N, Moita MA (2011). Time Determines the Neural Circuit Underlying Associative Fear Learning. Front Behav Neurosci.

[80] Rose TR, Fernandez de Velasco ME, Vo BN, Tipps ME, Wickman K (2021). Impact of Acute and Persistent Excitation of Prelimbic Pyramidal Neurons on Motor Activity and Trace Fear Learning. J Neurosci.

[81] Blum S, Runyan JD, Dash PK (2006). Inhibition of Prefrontal Protein Synthesis Following Recall Does Not Disrupt Memory for Trace Fear Conditioning. BMC Neurosci.

[82] Reis DS, Jarome TJ, Helmstetter FJ (2013). Memory formation for trace fear conditioning requires ubiquitin-proteasome mediated protein degradation in the prefrontal cortex. Front Behav Neurosci.

[83] Tang J, Ko S, Ding HK, Qiu CS, Calejesan AA, Zhuo M (2005). Pavlovian Fear Memory Induced by Activation in the Anterior Cingulate Cortex. Mol Pain.

[84] Zhao MG, Toyoda H, Lee YS, Wu LJ, Ko SW, Zhang XH (2005). Roles of NMDA NR2B Subtype Receptor in Prefrontal Long-Term Potentiation and Contextual Fear Memory. Neuron.

[85] Vetere G, Restivo L, Cole CJ, Ross PJ, Ammassari-Teule M, Josselyn SA (2011). Spine Growth in the Anterior Cingulate Cortex Is Necessary for the Consolidation of Contextual Fear Memory. Proc Natl Acad Sci USA.

[86] Einarsson EÖ, Nader K (2012). Involvement of the Anterior Cingulate Cortex in Formation, Consolidation, and Reconsolidation of Recent and Remote Contextual Fear Memory. Learn Memory.

[87] Bero AW, Meng J, Cho S, Shen AH, Canter RG, Ericsson M (2014). Early Remodeling of the Neocortex Upon Episodic Memory Encoding. Proc Natl Acad Sci USA.

[88] Xia F, Richards BA, Tran MM, Josselyn SA, Takehara-Nishiuchi K, Frankland PW (2017). Parvalbumin-Positive Interneurons Mediate Neocortical-Hippocampal Interactions That Are Necessary for Memory Consolidation. eLife.

[89] Frankland PW, Bontempi B, Talton LE, Kaczmarek L, Silva AJ (2004). The Involvement of the Anterior Cingulate Cortex in Remote Contextual Fear Memory. Science.

[90] Einarsson EÖ, Pors J, Nader K (2015). Systems Reconsolidation Reveals a Selective Role for the Anterior Cingulate Cortex in Generalized Contextual Fear Memory Expression. Neuropsychopharmacology.

[91] Goshen I, Brodsky M, Prakash R, Wallace J, Gradinaru V, Ramakrishnan C (2011). Dynamics of Retrieval Strategies for Remote Memories. Cell.

[92] Alvarez RP, Biggs A, Chen G, Pine DS, Grillon C (2008). Contextual fear conditioning in humans: cortical-hippocampal and amygdala contributions. J. Neurosci.

[93] Corodimas KP, LeDoux JE (1995). Disruptive Effects of Post-training Perirhinal Cortex Lesions on Conditioned Fear: Contributions of Contextual Cues. Behav Neurosci.

[94] Bucci DJ, Phillips RG, Burwell RD (2000). Contributions of postrhinal and perirhinal cortex to contextual information processing. Behav Neurosci.

[95] Kholodar-Smith DB, Boguszewski P, Brown TH (2008). Auditory Trace Fear Conditioning Requires Perirhinal Cortex. Neurobiol Learn Memory.

[96] Kholodar-Smith DB, Allen TA, Brown TH (2008). Fear Conditioning to Discontinuous Auditory Cues Requires Perirhinal Cortical Function. Behav Neurosci.

[97] Navaroli VL, Zhao Y, Boguszewski P, Brown TH (2012). Muscarinic Receptor Activation Enables Persistent Firing in Pyramidal Neurons from Superficial Layers of Dorsal Perirhinal Cortex. Hippocampus.

[98] Pitkänen A, Pikkarainen M, Nurminen N, Ylinen A (2000). Reciprocal Connections between the Amygdala and the Hippocampal Formation, Perirhinal Cortex, and Postrhinal Cortex in Rat. A Review. Ann NY Acad Sci.

[99] Egorov AV, Unsicker K, von Bohlen und Halbach O (2006). Muscarinic Control of Graded Persistent Activity in Lateral Amygdala Neurons. Eur J Neurosci.

[100] Baysinger AN, Kent BA, Brown TH (2012). Muscarinic Receptors in Amygdala Control Trace Fear Conditioning. PLoS One.

[101] Vetere G, Kenney JW, Tran LM, Xia F, Steadman PE, Parkinson J (2017). Chemogenetic Interrogation of a Brain-Wide Fear Memory Network in Mice. Neuron.

[102] Mount RA, Sridhar S, Hansen KR, Mohammed AI, Abdulkerim M, Kessel R (2021). Distinct Neuronal Populations Contribute to Trace Conditioning and Extinction Learning in the Hippocampal Ca1. eLife.

[103] Maren S (1999). Neurotoxic or Electrolytic Lesions of the Ventral Subiculum Produce Deficits in the Acquisition and Expression of Pavlovian Fear Conditioning in Rats. Behav Neurosci.

[104] Biedenkapp JC, Rudy JW (2009). Hippocampal and Extrahippocampal Systems Compete for Control of Contextual Fear: Role of Ventral Subiculum and Amygdala. Learn Memory.

[105] Dunn AR, Neuner SM, Ding S, Hope KA, O’Connell KMS, Kaczorowski CC. Cell-Type-Specific Changes in Intrinsic Excitability in the Subiculum Following Learning and Exposure to Novel Environmental Contexts. eNeuro. 2018;5: ENEURO.0484-18.2018. 10.1523/ENEURO.0484-18.2018 PMC6325565 30627661

[106] Liu D, Gu X, Zhu J, Zhang X, Han Z, Yan W (2014). Medial Prefrontal Activity During Delay Period Contributes to Learning of a Working Memory Task. Science.

