# Supplementary material for: Reactivation of encoding ensembles in the prelimbic cortex supports temporal associations
Source: Neuropsychopharmacology. 2024 Mar 7;49(8):1296–308. doi: 10.1038/s41386-024-01825-2 (PMC11224261; doi:10.1038/s41386-024-01825-2)
Supplement: Supplementary file 1 — Supplemental Material [file 41386_2024_1825_MOESM1_ESM.docx]

**Supplementary Figure 1 – Groups had similar freezing during habituation and training sessions and higher freezing after fear conditioning than before.** **(A)** In the opto-reactivation experiments, Generalized Linear Models (GZLM) did not show a significant effect of group in the freezing during the habituation (CFC-5s: W = 0.267; p = 0.605; CFC: W = 0.065; p = 0.799) or **(B)** the training (CFC-5s: W = 1.647; p = 0.199; CFC: W = 2.117; p = 0.146). **(C)** Freezing time during the light ON period was higher than the habituation in the opto-stimulation context. General Estimation Equations (GEE) showed a significant effect of the group (W = 6.701; p = 0.010), session (W = 67.513; p = 0.001), and interaction (W = 5,619; p =0.018) on the freezing of mice trained in the CFC-5s. LSD test for interaction showed that both ChR2 (p = 0.001; β = 1.092) and GFP (p = 0.001; β = 0.974) groups had higher freezing during the light ON than the habituation, and the ChR2 also than the GFP group during the ON period (p = 0.006; β = 0.885). GEE showed an effect of the session (W = 142.456; 0.001; β = 1.584) on the freezing in mice trained in the CFC. **(D)** In the opto-inhibition experiments, GZLM did not show a significant effect of the group on the freezing during the habituation (CFC-5s: W = 1.608; p = 0.205; CFC: W = 0.086; p = 0.769). **(E)** NpACY group had higher freezing than the GFP group in CFC-5s-trained mice (CFC-5s: W = 6.457; p = 0.011; CFC: W = 0.842; p = 0.359). Given its low magnitude, it probably does not represent a biological relevance. **(F)** Freezing during the light OFF was higher than during the training in the conditioned context. GEE showed a significant effect of the session on the freezing in CFC-5s- (W = 315.404; p = 0.001; β = 2.011) or CFC-trained mice (W = 341.199; p = 0.001; β = 1.845). **(E)** GZLM did not show a significant effect of the virus on the freezing in the habituation (W = 0.484; p = 0.487) or **(J)** the training (W = 0.028; p = 0.868). **(K)** Freezing during the ON period was higher than habituation in the opto-stimulation context (GZLM W = 48.597; p = 0.001; β = 1.791) and higher during the OFF period than the training in the conditioned context (GZLM W = 31.574; p = 0.001; β = 1.771). Mean freezing time (± standard error). Dots show sample distribution. ***** Indicates p < 0.050. **CFC:** contextual fear conditioning; **CFC-5s:** CFC with a 5-second interval; **CFC-5s DIF:** CFC-5s using different contexts as the CS and US background.

**Supplementary Figure 2 – All groups showed a similar percentage of infected cells. (A)** In the opto-reactivation experiments, Generalized Linear Models (GZLM) did not show a significant effect of the task (CFC-5s x CFC, W = 0.355; df = 1; p = 0.551), the virus (ChR2 x GFP, W = 2.568; df = 1; p = 0.109) or their interaction (W = 0.829; df = 1; p = 0.363) in the expression of GFP-positive cells. **(B)** In the silencing experiment, GZLM also did not show a significant effect of the task (CFC-5s x CFC, W = 1.651; df = 1; p = 0.199), the virus (NpACY x GFP, W = 1.592; df = 1; p = 0.207) or the interaction (W = 0.001; df = 1; p = 0.971). **(C)** Using the CFC-5s DIF task, GZLM did not show a significant effect of the virus (ChR2 x NpACY, W = 0.090; df = 1; p = 0.764) in the expression of GFP-positive cells. Mean (± standard error) of infected cells (GFP-positive cells) in the PL. * Indicates p < 0.050.

|  | | **Supplementary Table 1 – Number of cells in the regions** | | | | | | |
| --- | --- | --- | --- | --- | --- | --- | --- | --- |
| **Region** | **AP Coordinate** | | **CFC (1)** | **CFC-5s (2)** | **HC (3)** | **Wald** | **p-value** |  |
| **AC** | +1.98, 1.78, +1.54 | | 1400.45 ± 117.76 | 1413.89 ± 199.20 | 1190.53 ± 110.26 | 1.956 | 0.376 |  |
| **PL** |  |  | 1809.22 ± 127.73 | 1726.83 ± 174.19 | 1717.67 ± 85.41 | 0.353 | 0.838 |  |
| **IL** |  |  | 1848.43 ± 173.00 | 1802.50 ± 210.46 | 1481.25 ± 66.63 | 3.482 | 0.175 |  |
| **BLA** | -1.06, -1.34, -1.58 | | 901.40 ± 189.27 | 986.08 ± 184.90 | 835.58 ± 163.66 | 0.390 | 0.823 |  |
| **BMA** |  |  | 1459.85 ± 261.66 | 1674.00 ± 271.98 | 1365.50 ± 171.29 | 0.958 | 0.619 |  |
| **CEA** |  |  | 1504.70 ± 334.79 | 1570.06 ± 298 | 1496.33 ± 272.41 | 0.039 | 0.981 |  |
| **LA** |  |  | 685.20 ± 152.69 | 761.28 ± 155.92 | 722.42 ± 50.32 | 0.199 | 0.905 |  |
| **MEA** |  |  | 1880.21 ± 320.93 | 2080.36 ± 288.95 | 1854.42 ± 145.79 | 0.475 | 0.789 |  |
| **dCA1** | -1.58, -1.82, -2.06 | | 729.47 ± 86.56 | 823.86 ± 112.05 | 723.00 ± 107.04 | 0.717 | 0.699 |  |
| **dDG** |  |  | 1043.33 ± 147.90 | 1044.22 ± 120.72 | 996.67 ± 46.79 | 0.122 | 0.941 |  |
| **vCA1** | -3.40, -3.52, -3.64 | | 1491.38 ± 215.09 | 1583.47 ± 298.40 | 945.50 ± 198.81 | 5.356 | 0.069 |  |
| **vDG** |  |  | 938.92 ± 106.80 | 1038.08 ± 74.03 | 790.75 ± 127.44 | 3.128 | 0.209 |  |
| **vSUB** |  |  | 1206.45 ± 182.68 | 1427.93 ± 287.66 | 1374.08 ± 102.31 | 0.790 | 0.674 |  |
| **PER_35** | -2.06, -2.80, -3.64 | | 565.42 ± 93.40 | 614.92 ± 124.09 | 360.75 ± 68.45 | 4.322 | 0.115 |  |
| **PER_36** |  |  | 704.97 ± 113.90 | 788.22 ± 146.64 | 581.67 ± 90.07 | 1.591 | 0.451 |  |

**Supplementary Table 1 – Groups had a similar number of cells in the regions.** Anteroposterior (AP) coordinates from bregma (mm). Mean (± standard error) of the total number of cells (DAPI-positive cells). Generalized Linear Models. **AC**: anterior cingulate cortex; **BLA**: basolateral amygdala; **BMA**: basomedial amygdala; **CEA**: central amygdala; **CFC:** contextual fear conditioning; **CFC-5s:** CFC with a 5-second interval; **DAPI:** 4’,6-diamidino-2-phenylindole; **dCA1**: dorsal CA1; **dDG**: dorsal dentate gyrus; **HC:** homecage; **IL**: infralimbic cortex; **LA**: lateral amygdala; **MEA**: medial amygdala; **PER_35:** perirhinal cortex area 35; **PER_36:** perirhinal cortex area 36; **PL**: prelimbic cortex; **vCA1**: ventral CA1; **vDG**: ventral dentate gyrus; **vSUB**: ventral subiculum.

| **Supplementary Table 2 –**  **Linear Regression Models estimating the activity in test (response) by the activity in the CFC or CFC-5s training (predictor) in each region** | | | | | | | | | | | | | |
| --- | --- | --- | --- | --- | --- | --- | --- | --- | --- | --- | --- | --- | --- |
|  | | **CFC** | | | | | | **CFC-5s** | | | | | |
| **Region** | **r^2^** | | **B** | **95% CI** | **t** | **P-value** | **r^2^** | | **B** | **95% CI** | **t** | **P-value** |  |
| **AC** | **0.667** | | **0.164** | **0.031 - 0.297** | **3.168** | **0.025 *** | 0.024 | | -0.047 | -0.456 - 0.363 | -0.316 | 0.768 |  |
| **BLA** | **0.724** | | **0.226** | **0.066 - 0.387** | **3.620** | **0.015 *** | 0.053 | | 0.031 | -0.153 - 0.216 | 0.474 | 0.660 |  |
| **BMA** | **0.709** | | **0.240** | **0.063 - 0.417** | **3.492** | **0.017 *** | 0.475 | | 0.122 | -0.056 - 0.299 | 1.902 | 0.130 |  |
| **CEA** | 0.016 | | 0.006 | -0.049 - 0.061 | 0.285 | 0.787 | 0.008 | | -0.011 | -0.183 - 0.161 | -0.178 | 0.867 |  |
| **dCA1** | **0.674** | | **0.165** | **0.033 - 0.297** | **3.216** | **0.024 *** | 0.045 | | 0.349 | -1.894 - 2.593 | 0.432 | 0.688 |  |
| **dDG** | 0.004 | | -0.022 | -0.424 - 0.380 | -0.143 | 0.892 | 0.257 | | -0.368 | -1.236 - 0.501 | -1.175 | 0.305 |  |
| **IL** | 0.108 | | 0.185 | -0.380 - 0.711 | 0.779 | 0.471 | 0.056 | | 0.570 | -2.692 - 3.833 | 0.485 | 0.653 |  |
| **LA** | 0.301 | | 0.238 | -0.179 - 0.655 | 1.466 | 0.202 | 0.517 | | 0.159 | -0.054 - 0.372 | 2.071 | 0.107 |  |
| **MEA** | **0.589** | | **0.229** | **0.009 - 0.450** | **2.676** | **0.044 *** | 0.309 | | 0.225 | -0.242 - 0.692 | 1.337 | 0.252 |  |
| **PER_35** | **0.575** | | **0.391** | **0.005 - 0.777** | **2.602** | **0.048 *** | 0.061 | | -0.049 | -0.312 - 0.215 | -0.511 | 0.636 |  |
| **PER_36** | **0.841** | | **0.450** | **0.225 - 0.675** | **5.140** | **0.004 *** | 0.405 | | 0.225 | -0.153 - 0.604 | 1.651 | 0.174 |  |
| **PL** | **0.729** | | **0.303** | **0.091 - 0.515** | **3.671** | **0.014 *** | **0.746** | | **1.105** | **0.211 - 1.999** | **3.431** | **0.027 *** |  |
| **vCA1** | **0.648** | | **0.338** | **0.052 - 0.624** | **3.036** | **0.029 *** | 0.307 | | -0.828 | -2.556 - 0.899 | -1.331 | 0.254 |  |
| **vDG** | **0.629** | | **0.628** | **0.074 - 1.182** | **2.913** | **0.033 *** | 0.025 | | -0.134 | -1.290 - 1.023 | -0.321 | 0.764 |  |
| **vSUB** | 0.513 | | 0.411 | -0.049 - 0.872 | 2.296 | 0.070 | 0.001 | | -0.009 | -0.613 - 0.594 | -0.440 | 0.967 |  |

**Supplementary Table 2 – Td expression following the CFC training predicted the c-Fos expression following the test in the AC, BLA, BMA, dCA1, MEA, PER_35, PER_36, PL, vCA1, and vSUB. Td expression following CFC-5s training predicted the c-Fos expression following the test in the PL.** Linear Regression Models for CFC and CFC-5s groups in each region. * Indicates p < 0.050. **r^2^**: r-squared goodness-of-fit measure; **B:** beta coefficient; **95% CI**: 95% confidence interval of the r^2^; **t:** Linear Regression t-test; **AC**: anterior cingulate cortex; **BLA**: basolateral amygdala; **BMA**: basomedial amygdala; **CEA**: central amygdala; **CFC:** contextual fear conditioning; **CFC-5s:** CFC with a 5-second interval; **dCA1**: dorsal CA1; **dDG**: dorsal dentate gyrus; **IL**: infralimbic cortex; **LA**: lateral amygdala; **MEA**: medial amygdala; **PER_35:** perirhinal cortex area 35; **PER_36:** perirhinal cortex area 36; **PL**: prelimbic cortex; **vCA1**: ventral CA1; **vDG**: ventral dentate gyrus; **vSUB**: ventral subiculum.


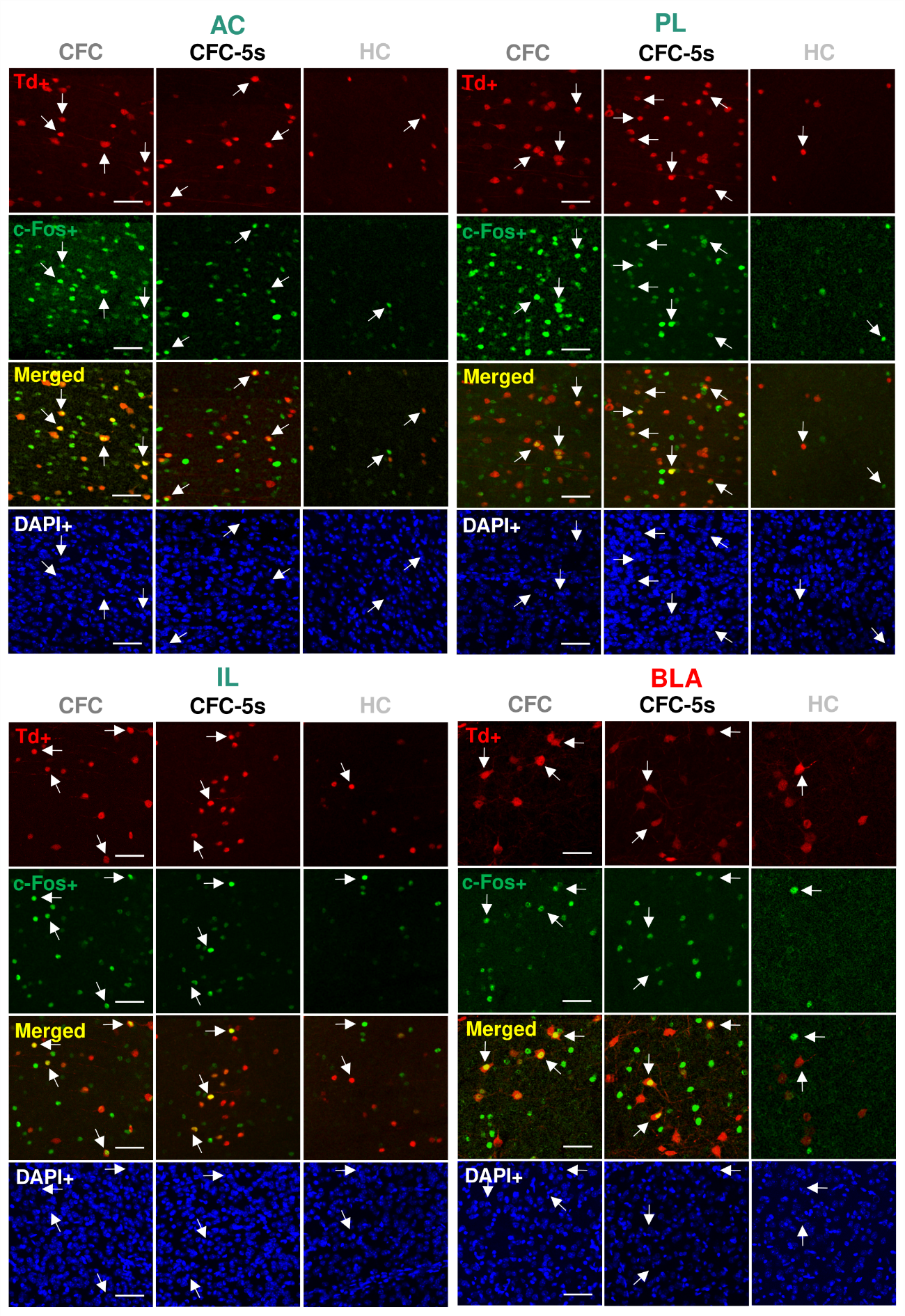
**Supplementary Figure 3 –** Representative images (magnified view) of positive cells to **TdTomato** (red), **c-fos** (green), **DAPI**, 4’,6-diamidino-2-phenylindole (blue), and double-labeled cells (Td- and c-Fos-positive) in the cingulate (**AC**), prelimbic (**PL**), and infralimbic (**IL**) cortices and basolateral amygdala (**BLA**) in the contextual fear conditioning (**CFC**), CFC with a 5-second interval (**CFC-5s**) and homecage (**HC**) groups. White arrows indicate positive cells. Scale bar = 100 μm.


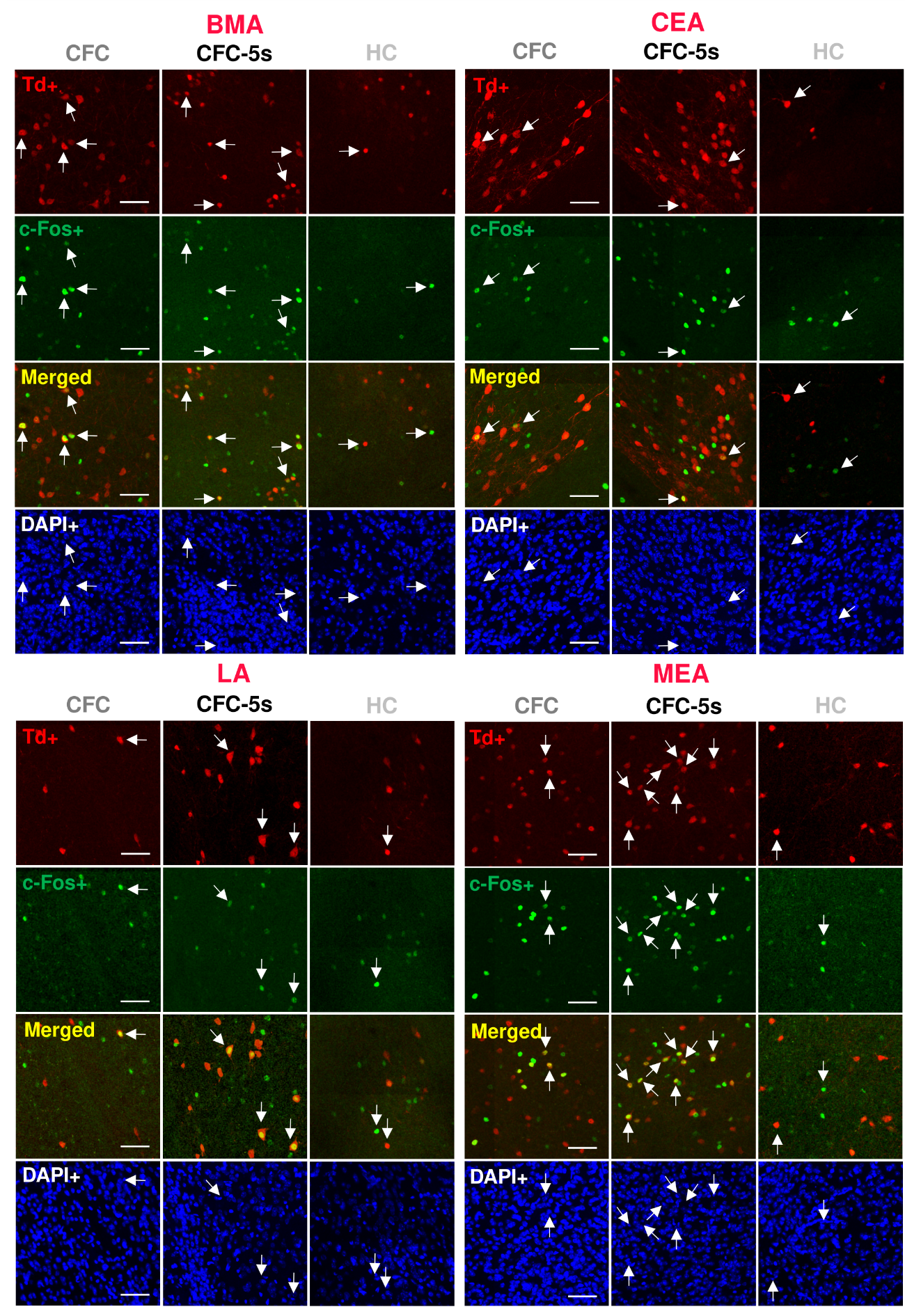
**Supplementary Figure 4 –** Representative images (magnified view) of positive cells to **TdTomato** (red), **c-fos** (green), **DAPI**, 4’,6-diamidino-2-phenylindole (blue), and double-labeled cells (Td- and c-Fos-positive) in the basomedial (**BMA**), central (**CEA**), lateral (**LA**), and medial (**MEA**) amygdala in the contextual fear conditioning (**CFC**), CFC with a 5-second interval (**CFC-5s**) and homecage (**HC**) groups. White arrows indicate positive cells. Scale bar = 100 μm.

**
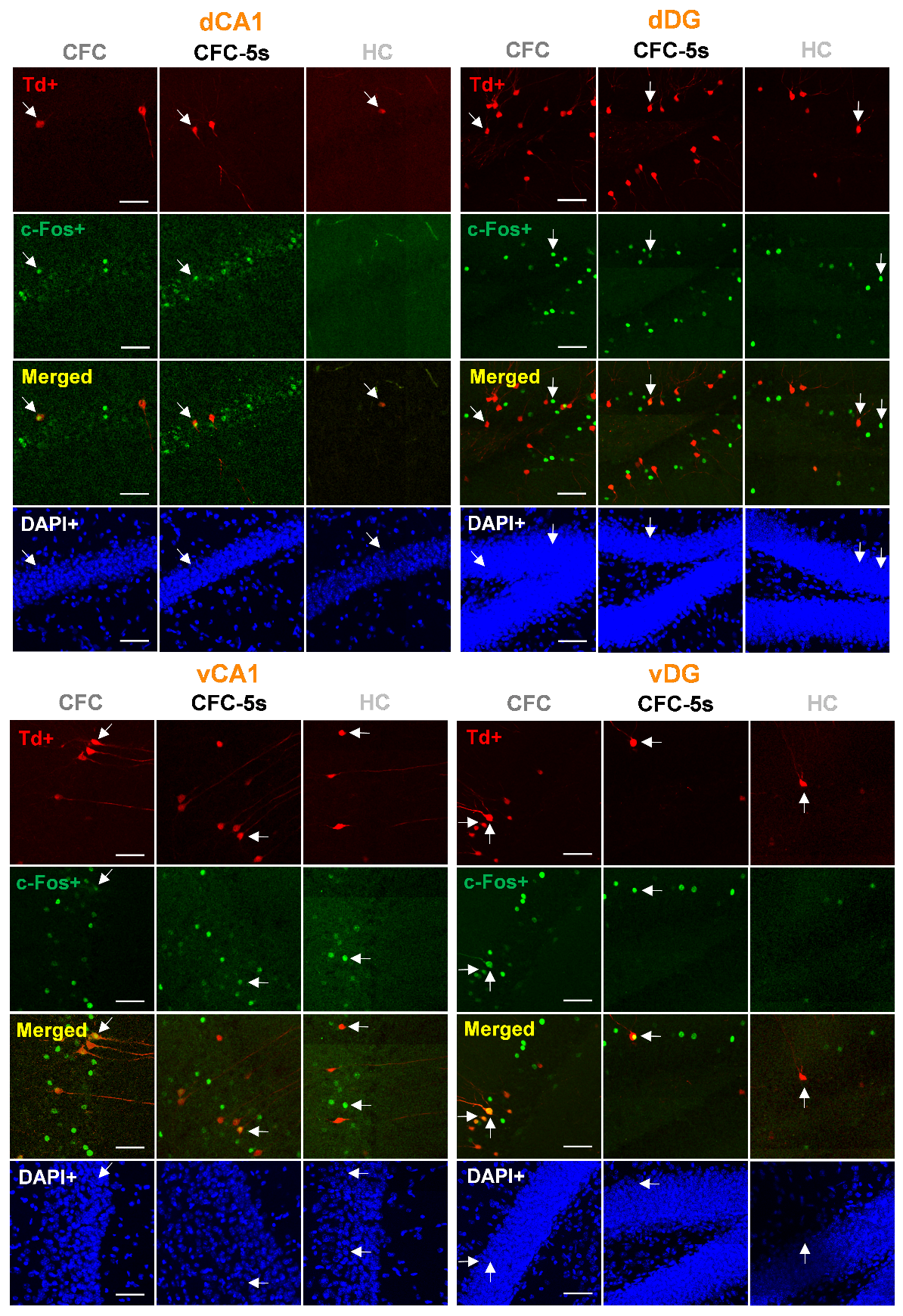
Supplementary Figure 5 –** Representative images (magnified view) of positive cells to **TdTomato** (red), **c-fos** (green), **DAPI**, 4’,6-diamidino-2-phenylindole (blue), and double-labeled cells (Td- and c-Fos-positive) in the dorsal CA1 (**dCA1**), dorsal dentate gyrus (**dDG**), ventral CA1 (**vCA1**), and ventral dentate gyrus (**vDG**) the contextual fear conditioning (**CFC**), CFC with a 5-second interval (**CFC-5s**) and homecage (**HC**) groups. White arrows indicate positive cells. Scale bar = 100 μm.

**Supplementary Figure 6 –** Representative images (magnified view) of positive cells to **TdTomato** (red), **c-fos** (green), **DAPI**, 4’,6-diamidino-2-phenylindole (blue), and double-labeled cells (Td- and c-Fos-positive) in the ventral subiculum (**vSUB**), perirhinal cortex area 35 (**PER_35**), and perirhinal cortex area 36 (**PER_36**) in the contextual fear conditioning (**CFC**), CFC with a 5-second interval (**CFC-5s**) and homecage **(HC**) groups. White arrows indicate positive cells. Scale bar = 100 μm.

| **Supplementary Table 3 – Comparison of mean correlation coefficients within or between major brain subdivisions** | | | | | | | | | | | |
| --- | --- | --- | --- | --- | --- | --- | --- | --- | --- | --- | --- |
|  | **CFC (1)** | **CFC-5s (2)** | **Wald** | **P-value** | **β** |  | **CFC (1)** | **CFC-5s (2)** | **Wald** | **P-value** | **β** |
| **Within anatomical groups:** | | | | | | **Between anatomical groups:** | | | | | |
| **mPFC** | 0.819 ± 0.107 | 0.951 ± 0.023 | 1.730 | 0.188 | 0.680 | **mPFC** | -0.091 ± 0.078 | -0.072 ± 0.063 | 0.045 | 0.832 | 0.050 |
| **AMY** | 0.787 ± 0.067 | 0.894 ± 0.032 | 2.267 | 0.132 | 0.521 | **AMY** | **0.007 ± 0.052** | **0.295 ± 0.057** | **14.271** | **0.001 ^2^** | **0.703** |
| **HPC** | 0.631 ± 0.126 | 0.418 ± 0.218 | 0.795 | 0.372 | 0.381 | **HPC** | 0.236 ± 0.060 | 0.116 ± 0.064 | 1.969 | 0.161 | 0.294 |
| **PH** | 0.694 ± 0.088 | 0.572 ± 0.151 | 0.556 | 0.456 | 0.336 | **PH** | 0.333 ± 0.059 | 0.320 ± 0.065 | 0.022 | 0.883 | 0.032 |
| **Between mPFC and:** | | | | | | **Between AMY and:** | | | | | |
| **AMY** | **-0.342 ± 0.094** | **-0.025 ± 0.067** | **8.120** | **0.012 ^2^** | **0.908** | **HPC** | 0.165 ± 0.053 | 0.272 ± 0.084 | 1.216 | 0.324 | 0.339 |
| **HPC** | 0.029 ± 0.145 | -0.191 ± 0.109 | 1.615 | **0.306** | 0.492 | **PH** | **0.146 ± 0.071** | **0.646 ± 0.065** | **28.641** | **0.006 ^2^** | **1.374** |
| **PH** | 0.164 ± 0.135 | 0.009 ± 0.105 | 0.919 | 0.338 | 0.428 |  |  |  |  |  |  |
|  |  |  |  |  |  |  |  |  |  |  |  |
| **Between HPC and:** | | | | | |  |  |  |  |  |  |
| **PH** | **0.562 ± 0.087** | **0.162 ± 0.115** | **8.366** | **0.008 ^1^** | **0.995** |  |  |  |  |  |  |

**Supplementary Table 3 – CFC-5s had a significantly higher correlation between the amygdala and the parahippocampal area and CFC between the hippocampus and the parahippocampal area.** Mean (± standard error) of the Pearson’s correlation coefficient within each anatomical group, between one anatomical group and the remaining ones, or between pairs of anatomical groups in CFC and CFC-5s groups. Generalized Linear Models using False Discovery Rate to correct for multiple comparisons (adjusted p-values). **^1^** Indicates adjusted p < 0.050, with a higher absolute mean correlation coefficient in the CFC group; **^2^** indicates p < 0.050, with a higher absolute mean correlation coefficient in the CFC-5s group. Standardized betas (β) were used as a measure of effect size. **AMY**: amygdala nuclei (includes basolateral, basomedial, central, lateral, and medial amygdala nuclei); **HPC:** hippocampus (includes dorsal and ventral dentate gyrus, ventral and dorsal CA1); **mPFC:** the medial prefrontal cortex (includes cingulate, prelimbic and infralimbic cortices); **PH:** parahippocampal area (has ventral subiculum, perirhinal cortex area 35 and 36). **CFC:** contextual fear conditioning; **CFC-5s:** CFC with a 5-second interval.

| **Supplementary Table 4 – Correlation coefficients differences between the CFC-5s and CFC groups** | | | | | | | | | | | | | | | |
| --- | --- | --- | --- | --- | --- | --- | --- | --- | --- | --- | --- | --- | --- | --- | --- |
|  | **AC** | **PL** | **IL** | **BLA** | **BMA** | **CEA** | **LA** | **MEA** | **dCA1** | **dDG** | **vCA1** | **vDG** | **vSUB** | **PER_35** | **PER_36** |
| **AC** | NA  NA | 0.510  0.067 | 0.294  0.100 | 0.560  0.133 | 0.268  0.500 | 0.941  0.067 | 0.057  0.883 | 0.279  0.550 | 0.474  0.183 | 0.075  0.850 | 0.281  0.433 | 0.416  0.417 | 0.139  0.633 | 0.317  0.583 | 0.020  0.950 |
| **PL** | 0.510  0.067 | NA  NA | 0.014  0.817 | 0.147  0.767 | 0.064  0.850 | 0.562  0.350 | 0.181  0.800 | 0.179  0.750 | 0.175  0.717 | 0.406  0.233 | 0.321  0.483 | **1.212**  **0.001^1^** | 0.573  0.150 | 0.347  0.550 | 0.338  0.417 |
| **IL** | 0.294  0.100 | 0.014  0.817 | NA  NA | 0.529  0.333 | 0.191  0.617 | 0.906  0.100 | 0.057  0.967 | 0.435  0.450 | 0.263  0.617 | 0.320  0.500 | 0.011  0.983 | 0.883  0.067 | 0.388  0.383 | 0.067  0.883 | 0.248  0.533 |
| **BLA** | 0.560  0.133 | 0.147  0.767 | 0.529  0.333 | NA  NA | 0.083  0.317 | 0.169  0.150 | 0.563  0.067 | 0.193  0.217 | 0.242  0.567 | 0.033  0.917 | 0.200  0.583 | 0.590  0.200 | 0.305  0.368 | 0.765  0.150 | **0.975**  **0.017^2^** |
| **BMA** | 0.268  0.500 | 0.064  0.850 | 0.191  0.617 | 0.083  0.317 | NA  NA | 0.321  0.083 | 0.236  0.300 | 0.066  0.617 | 0.389  0.383 | 0.222  0.483 | 0.531  0.317 | 0.814  0.083 | 0.111  0.767 | 0.734  0.217 | **0.904**  **0.050^2^** |
| **CEA** | 0.941  0.067 | 0.562  0.350 | 0.906  0.100 | 0.169  0.150 | 0.321  0.083 | NA  NA | 0.620  0.083 | 0.270  0.183 | 0.608  0.217 | 0.140  0.817 | 0.088  0.833 | 0.380  0.400 | 0.031  0.900 | 0.808  0.133 | 0.850  0.350 |
| **LA** | 0.057  0.883 | 0.181  0.800 | 0.057  0.967 | 0.563  0.067 | 0.236  0.300 | 0.620  0.083 | NA  NA | 0.370  0.150 | 0.431  0.283 | 0.083  0.917 | 0.403  0.533 | 0.087  0.817 | 0.004  0.967 | 0.679  0.133 | 0.402  0.100 |
| **MEA** | 0.279  0.550 | 0.179  0.750 | 0.435  0.450 | 0.193  0.217 | 0.066  0.617 | 0.270  0.183 | 0.370  0.150 | NA  NA | 0.169  0.650 | 0.172  0.717 | 0.324  0.600 | 0.520  0.333 | 0.023  0.950 | 0.652  0.150 | 1.099  0.083 |
| **dCA1** | 0.474  0.183 | 0.175  0.717 | 0.263  0.617 | 0.242  0.567 | 0.389  0.383 | 0.608  0.217 | 0.431  0.283 | 0.169  0.650 | NA  NA | 0.408  0.367 | **0.887**  **0.050^1^** | 0.502  0.400 | **1.157**  **0.001^1^** | 0.786  0.133 | 0.331  0.533 |
| **dDG** | 0.075  0.850 | 0.406  0.233 | 0.320  0.500 | 0.033  0.917 | 0.222  0.483 | 0.140  0.817 | 0.083  0.917 | 0.172  0.717 | 0.408  0.367 | NA  NA | 0.343  0.283 | **0.888**  **0.033^1^** | **0.943**  **0.033^1^** | 0.737  0.150 | 0.044  0.083 |
| **vCA1** | 0.281  0.433 | 0.321  0.483 | 0.011  0.983 | 0.200  0.583 | 0.531  0.317 | 0.088  0.833 | 0.403  0.533 | 0.324  0.600 | **0.887**  **0.050^1^** | 0.343  0.283 | NA  NA | 0.083  0.650 | 0.597  0.083 | 0.005  0.950 | 0.144  0.783 |
| **vDG** | 0.416  0.417 | **1.212**  **0.001^1^** | 0.883  0.067 | 0.590  0.200 | 0.814  0.083 | 0.380  0.400 | 0.087  0.817 | 0.520  0.333 | 0.502  0.400 | **0.888**  **0.033^1^** | 0.083  0.650 | NA  NA | 0.353  0.117 | 0.312  0.117 | 0.444  0.167 |
| **vSUB** | 0.139  0.633 | 0.573  0.150 | 0.388  0.383 | 0.305  0.368 | 0.111  0.767 | 0.031  0.900 | 0.004  0.967 | 0.023  0.950 | **1.157**  **0.001^1^** | **0.943**  **0.033^1^** | 0.597  0.083 | 0.353  0.117 | NA  NA | 0.603  0.100 | 0.310  0.417 |
| **PER_35** | 0.317  0.583 | 0.347  0.550 | 0.067  0.883 | 0.765  0.150 | 0.734  0.217 | 0.808  0.133 | 0.679  0.133 | 0.652  0.150 | 0.786  0.133 | 0.737  0.150 | 0.005  0.950 | 0.312  0.117 | 0.603  0.100 | NA  NA | 0.361  0.283 |
| **PER_36** | 0.020  0.950 | 0.338  0.417 | 0.248  0.533 | **0.975**  **0.017^2^** | **0.904**  **0.050^2^** | 0.850  0.350 | 0.402  0.100 | 1.099  0.083 | 0.331  0.533 | 0.044  0.083 | 0.144  0.783 | 0.444  0.167 | 0.310  0.417 | 0.361  0.283 | NA  NA |

**Supplementary Table 4 – CFC-5s had a significantly higher negative correlation between the PL and vDG and a significantly higher positive correlation between the PER_36 and BLA and BMA, whereas the CFC had a higher positive correlation between the dCA1 and vCA1, the dDG and vDG, and the vSUB and the dCA1 and dDG.** Upper panels show the observed correlation coefficient differences between the CFC-5s and CFC groups for each pairwise of regions; lower panels show the p-values of the permutation tests (calculated as the frequency of correlation coefficient differences in the resampling matrixes that were higher than the observed in the original matrixes, from the 1000 resampling performed). **^1^** Indicates p < 0.050, with a higher correlation coefficient in the CFC group; **^2^** indicates p < 0.050, with a higher correlation coefficient in the CFC-5s group. **AC**: anterior cingulate cortex; **BLA**: basolateral amygdala; **BMA**: basomedial amygdala; **CEA**: central amygdala; **dCA1**: dorsal CA1; **dDG**: dorsal dentate gyrus; **IL**: infralimbic cortex; **LA**: lateral amygdala; **MEA**: medial amygdala; **NA:** not applicable; **PER_35**: perirhinal cortex area 35; **PER_36:** perirhinal cortex area 36; **PL**: prelimbic cortex; **vCA1**: ventral CA1; **vDG**: ventral dentate gyrus; **vSUB**: ventral subiculum.

**Supplementary Figure 7 – The mean reactivation did not predict or correlate with the strength of the correlation coefficients. (A)** Linear Regression Models did not show a significant effect of double-labeled cells (Td- and c-Fos-positive) in predicting the Pearson’s correlation coefficients (correlation of double-labeled cells) in thirteen region, except the AC and IL (AC: r^2^ = 0.397; B = 1.047; t = 2.690; p = 0.021; PL: r^2^ = 0.060; B = 0.063; t = 0.839; p = 0.420; IL: r^2^ = 0.314; B = 0.771; t = 2.245; p = 0.046; BLA: r^2^ = 0.001; B = 0.015; t = 0.047; p = 0.963; BMA: r^2^ = 0.036; B = 0.139; t = 0.643; p = 0.534; CEA: r^2^ = 0.062; B = -0.517; t = -0.850; p = 0.413; LA: r^2^ = 0.079; B = -0.291; t = -1.011; p = 0.332; MEA: r^2^ = 0.081; B = -0.141; t = -0.983; p = 0.347; dCA1: r^2^ = 0.001; B = -0.070; t = -0.116; p = 0.910; dDG: r^2^ = 0.001; B = 0.015; t = 0.015; p = 0.988; vCA1: r^2^ = 0.019; B = 0.248; t = 0.459; p = 0.655; vDG: r^2^ = 0.101; B = -0.684; t = -1.111; p = 0.290; vSUB: r^2^ = 0.001; B = 0.024; t = 0.046; p = 0.964; PER_35: r^2^ = 0.090; B = -0.190; t = -1.041; p = 0.320; PER_36: r^2^ = 0.132; B = -0.180; t = -1.296; p = 0.222). The mean of double-labeled cells was not significantly correlated with the mean r^2^ (p = 0.848). **(B)** The mean of double-labeled cells was also not correlated with the mean of the correlation coefficients (p = 0.977). **r:** Pearson’s correlation coefficient; **r^2^**: r-squared goodness-of-fit measure; **B:** beta coefficient **t:** Linear Regression t-test; **AC:** anterior cingulate cortex; **BLA:** basolateral amygdala; **BMA:** basomedial amygdala; **CEA:** central amygdala; **dCA1**: dorsal CA1; **dDG:** dorsal dentate gyrus; **IL:** infralimbic cortex; **LA:** lateral amygdala; **MEA:** medial amygdala; **PER_35:** perirhinal cortex area 35; **PER_36:** perirhinal cortex area 36; **PL:** prelimbic cortex; **vCA1:** ventral CA1; **vDG:** ventral dentate gyrus; **vSUB:** ventral subiculum.

.

| **Supplementary Table 5 – Overview of the CFC-5s and CFC networks** | | | | | | | | |
| --- | --- | --- | --- | --- | --- | --- | --- | --- |
| **Measure** | **CFC Positive** | **CFC-5s Positive** | **GZLM Wald** | **GZLM p-value** | **CFC Negative** | **CFC-5s Negative** | **GZLM Wald** | **GZLM p-value** |
| **Nodes** | 15 | 15 | NA | NA | 15 | 14 | NA | NA |
| **Edges** | 72 | 72 | NA | NA | 33 | 33 | NA | NA |
| **Connected Components** | 1 | 1 | NA | NA | 1 | 2 | NA | NA |
| **Graph Density** | 0.686 | 0.686 | NA | NA | 0.314 | 0.314 | NA | NA |
| **Network Diameter** | 3 | 2 | NA | NA | 3 | 3 | NA | NA |
| **Average Path Length** | 1.343 | 1.314 | NA | NA | 1.781 | 1.703 | NA | NA |
| **Modularity** | 0.243 | 0.185 | NA | NA | -0.119 | -0.102 | NA | NA |
| **Local Efficiency** | 0.856 ± 0.005 | 0.866 ± 0.007 | 1.819 | 0.177 | 0.705 ± 0.018 | 0.746 ± 0.018 | 2.964 | 0.085 |
| **Global**  **Efficiency** | 0.838 ± 0.024 | 0.843 ± 0.025 | 0.021 | 0.885 | 0.641 ± 0.024 | 0.626 ± 0.053 | 0.760 | 0.383 |
| **Average Cluster Coefficient** | 0.816 ± 0.025 | 0.818 ± 0.031 | 0.002 | 0.962 | 0.222 ± 0.108 | 0.254 ± 0.092 | 0.060 | 0.807 |
| **Average Degree** | 19.200 ± 1.209 | 19.200 ±  1.397 | 0.000 | 1.000 | 8.800 ± 1.209 | 8.800 ± 1.450 | 0.000 | 1.000 |
| **Average Weighted Degree** | 8.591 ±  0.598 | 9.833 ± 1.158 | 1.043 | 0.307 | -2.830 ± 0.691 | -2.668 ± 0.554 | 0.039 | 0.843 |

**Supplementary Table 5 – CFC and CFC-5s co-reactivation (positive or negative weights) networks had similar topological measures in between-group comparisons**. **Nodes:** number of regions; **edges:** number of correlation coefficients; **connected components:** number of subgraphs in which all pairs of nodes are connected via a path (edge); **graph density:** how many edges the network has, from the total of possible ones (a complete network would have graph density of 1); **network diameter:** the longest distance (sum of edges) between any two nodes; **average path length:** the network average distance (number of edges) between all pair of nodes; **modularity:** the quality of the partition of nodes into communities, ranging from -1 to +1; **average degree:** the average number of edges (connections) per node (region); **average weighted degree:** the average degree pondered by the weight of the correlation coefficient; **global efficiency:** the network average of the nodal efficiencies of all nodes (computed for each node as the inverse of the harmonic mean of the shortest path length, the minimum number of edges, between the node and all the others). Includes disconnected nodes; **local efficiency:** the network average of the nodal efficiencies of the neighbors of a node, excluding the node itself; **nodal efficiency:** the network average of the harmonic mean of the shortest path length (minimum number of edges) between the node and all the other nodes in the network; **average cluster coefficient**: the network average of the number of neighbors connected, from the total number of possible connections among the neighbors of a node (*i.e.,* number of connected triangles from the possible ones. Data are shown in mean (± standard error). Generalized Linear Models (**GZLM**). **CFC:** contextual fear conditioning; **CFC-5s:** CFC with a 5-second interval; **NA:** GZLM not applicable.

**Supplementary Figure 8 – CFC-5s and CFC co-reactivation networks had a similar distribution of correlation coefficient strength, an edge measure.** Cumulative distributions of the standardized Pearson’s correlation coefficients (**Fisher’s Z**) in the CFC (grey circles) and CFC-5s (black circles) networks using positive **(A)** or negative **(B)** correlation coefficients to build the networks. Two-sample Kolmogorov-Smirnov test; p = 0.131 in **(A)** and p = 0.843 in **(B). CFC:** contextual fear conditioning; **CFC-5s:** CFC with a 5-second interval.

**Supplementary Figure 9 – Co-reactivation (negative weight) networks of (A) CFC-5s** **and** **(B)** **CFC**. Nodes represent regions. Colors indicate the node’s anatomical group (scale, right). Edges between nodes represent negative correlation coefficients. Edge thickness and grey scale are proportional to the correlation strength. **(C, E)** Regions ranked in decrescent order of strength (**Str**), closeness (**Clo**), eigenvector (**Eig**), and betweenness (**Bet**). **(C, F)** The upper 25% of regions in three or more centrality measures were considered hubs (inside the red perimeter). **(G)** Centrality differences between CFC-5s and CFC groups. * Indicates significant centrality differences (p < 0.050) in the permutation test (calculated as the frequency of the centrality differences in the resampling networks higher than the observed centrality differences, from the 1000 resampling performed; p = resampling difference > empirical difference/1000). See all p-values in Supplementary Table 6. **(H)** Hubs of CFC-5s and CFC networks were intersected to identify stable hubs in fear conditioning (inside the red perimeter). **AC:** anterior cingulate cortex; **BLA:** basolateral amygdala; **BMA:** basomedial amygdala; **CEA:** central amygdala; **CFC:** contextual fear conditioning; **CFC-5s:** CFC with a 5-second interval; **dCA1**: dorsal CA1; **dDG:** dorsal dentate gyrus; **IL:** infralimbic cortex; **LA:** lateral amygdala; **MEA:** medial amygdala; **PER_35:** perirhinal cortex area 35; **PER_36:** perirhinal cortex area 36; **PL:** prelimbic cortex; **vCA1:** ventral CA1; **vDG:** ventral dentate gyrus; **vSUB:** ventral subiculum.

| **Supplementary Table 6 – Comparison of centrality measures between the CFC-5s and CFC networks** | | | | | | | | |
| --- | --- | --- | --- | --- | --- | --- | --- | --- |
| **Measure** | **Strength** | | **Closeness** | | **Eigenvector** | | **Betweenness** | |
| **Network** | **Positive** | **Negative** | **Positive** | **Negative** | **Positive** | **Negative** | **Positive** | **Negative** |
| **AC** | 0.400 | 0.400 | 0.200 | 0.250 | 0.250 | 0.250 | 0.250 | 0.150 |
| **BLA** | 0.100 | 0.300 | 0.150 | 0.250 | 0.300 | 0.200 | 0.150 | 0.200 |
| **BMA** | 0.200 | 0.800 | 0.200 | 0.750 | 0.900 | 0.600 | 0.350 | 0.650 |
| **CEA** | 0.150 | 0.200 | **0.050 ^2^** | 0.250 | 0.100 | 0.050 | 0.150 | **0.000 ^1^** |
| **dCA1** | 0.200 | 1.000 | 0.450 | 0.500 | 0.350 | 0.300 | 0.650 | 0.300 |
| **dDG** | 0.650 | 0.150 | 0.300 | 0.200 | 0.250 | 0.300 | 0.350 | 0.200 |
| **PER_35** | 0.250 | 0.800 | 0.750 | 1.000 | 0.700 | 0.900 | 1.000 | 0.300 |
| **PER_36** | 0.150 | 0.250 | **0.050 ^2^** | **0.050 ^1^** | 0.150 | 0.100 | 0.300 | 0.450 |
| **IL** | 1.000 | 0.850 | 0.900 | 0.700 | 0.850 | 0.800 | 0.750 | 0.500 |
| **LA** | **0.050 ^2^** | 0.950 | 0.850 | 0.750 | 0.750 | 0.850 | 1.000 | 0.950 |
| **MEA** | **0.001 ^2^** | 0.450 | 0.700 | 0.900 | 0.700 | 0.700 | 0.650 | 0.300 |
| **PL** | 0.300 | 0.750 | 0.750 | 0.600 | 0.600 | 0.600 | 0.500 | 0.450 |
| **vCA1** | 0.850 | 0.650 | 0.500 | 0.200 | 0.800 | 0.300 | 0.400 | 0.950 |
| **vDG** | 0.950 | 0.000 | 0.150 | 0.200 | 0.200 | 0.150 | 0.200 | 0.550 |
| **vSUB** | 0.100 | **0.050 ^2^** | **0.000 ^1^** | **0.001 ^2^** | 0.100 | 0.000 | 0.250 | 0.350 |

**Supplementary Table 6 –** **The CEA, LA, MEA, and PER_36 had higher centralities in the positive weight CFC-5s network, and the vDG and vSUB in the negative weight CFC-5s network. The vSUB had higher centrality in the positive weight CFC network, and the CEA and PER_36 in the negative weight CFC network.** P-values of the permutation test (calculated as the frequency of centrality differences in the resampling networks higher than the observed in the original networks from the 1000 resampling performed). **^1^** Indicates p < 0.050 and higher centrality in the CFC network; **^2^** Indicates p < 0.050 and higher centrality in the CFC-5s network. **AC:** anterior cingulate cortex; **BLA:** basolateral amygdala; **BMA:** basomedial amygdala; **CEA:** central amygdala; **CFC:** contextual fear conditioning; **CFC-5s:** CFC with a 5-second interval; **dCA1**: dorsal CA1; **dDG:** dorsal dentate gyrus; **IL:** infralimbic cortex; **LA:** lateral amygdala; **MEA:** medial amygdala; **NA:** not applicable; **PER_35:** perirhinal cortex area 35; **PER_36:** perirhinal cortex area 36; **PL:** prelimbic cortex; **vCA1:** ventral CA1; **vDG:** ventral dentate gyrus; **vSUB:** ventral subiculum.

| **Supplementary Table 7 – Comparison of topological properties in networks without hubs or non-hubs regions** | | | | | | | | |
| --- | --- | --- | --- | --- | --- | --- | --- | --- |
|  | **CFC networks** | | | | **CFC-5s networks** | | | |
| **Measure** | **without hubs** | **without non-hubs** | **GZLM Wald** | **GZLM p-value** | **without hubs** | **without non-hubs** | **GZLM Wald** | **GZLM p-value** |
| **Local Efficiency** | 0.815 ± 0.005 | 0.907 ± 0.005 | 191.807 | 0.001 | 0.817 ± 0.010 | 0.877 ± 0.010 | 19.631 | 0.001 |
| **Global**  **Efficiency** | 0.801 ± 0.021 | 0.891 ± 0.020 | 9.804 | 0.002 | 0.795 ± 0.026 | 0.840 ± 0.035 | 1477.230 | 0.290 |
| **Average Cluster Coefficient** | 0.774 ± 0.038 | 0.852 ± 0.026 | 3.180 | 0.075 | 0.762 ± 0.047 | 0.889 ± 0.028 | 5.216 | 0.022 |

**Supplementary Table 7 – Deleting hubs decreased network measures of integration and segregation.** We rebuild the CFC-5s and CFC positive weight network without its exclusive hubs (CFC-5s: CEA and PER_36; CFC: vSUB and vDG) or without its lowest centrality nodes (CFC-5s: AC and dDG; CFC: AC and CEA). Generalized Linear Models (GZLM) compared the local and global efficiency and average cluster coefficient between CFC-5s or CFC networks without hubs or non-hubs regions. Mean (± standard error). **AC:** anterior cingulate cortex; **CEA:** central amygdala; **CFC:** contextual fear conditioning; **CFC-5s:** CFC with a 5-second interval; **PER_36:** perirhinal cortex area 36; **vDG:** ventral dentate gyrus; **vSUB:** ventral subiculum.
